# Lipid nanoparticles as adjuvant of norovirus VLP vaccine augment cellular and humoral immune responses in a TLR9- and type I IFN-dependent pathway

**DOI:** 10.1128/jvi.01699-24

**Published:** 2024-11-04

**Authors:** Weiqian Dai, Man Xing, Lingjin Sun, Lihui Lv, Xiang Wang, Yihan Wang, Xueyang Pang, Yingying Guo, Jiling Ren, Dongming Zhou

**Affiliations:** 1 Department of Pathogen Biology, Basic Medical College, Tianjin Medical University, Tianjin, China; 2 Shanghai Public Health Clinical Center, Fudan University, Shanghai, China; University of Michigan Medical School, Ann Arbor, Michigan, USA

**Keywords:** norovirus, virus-like particles, lipid nanoparticles, adjuvants, type I IFN, immune responses

## Abstract

Norovirus (NoV) virus-like particles (VLPs) adjuvanted with aluminum hydroxide (Alum) are common vaccine candidates in clinical studies. Alum adjuvants usually inefficiently assist recombinant proteins to induce cellular immune responses. Thus, novel adjuvants are required to develop NoV vaccines that could induce both efficient humoral and robust cellular immune responses. Lipid nanoparticles (LNPs) are well-known mRNA delivery vehicles. Increasing evidence suggests that LNPs may have intrinsic adjuvant activity and can be used as adjuvants for recombinant protein vaccines; however, the underlying mechanism remains poorly understood. In this study, we compared the adjuvant effect of LNPs and Alum for a bivalent GI.1/GII.4 NoV VLP vaccine. Compared with Alum, LNP-adjuvanted vaccines induced earlier production of binding, blocking, and neutralizing antibodies and promoted a more balanced IgG2a/IgG1 ratio. It is crucial that LNP-adjuvanted vaccines induced stronger Th1-type cytokine-producing CD4^+^ T cell and CD8^+^ T cell responses than Alum. The adjuvant activity of LNPs depended on the ionizable lipid components. Mechanistically, LNPs activated innate immune responses in a type I IFN-dependent manner and were partially dependent on Toll-like receptor (TLR) 9, thus affecting the adaptive immune responses of the vaccine. This conclusion was supported by RNA-seq analysis and *in vitro* cell experiments and by the deeply blunted T cell responses in IFNαR1^−/−^ mice immunized with LNP-adjuvanted vaccines. This study not only identified LNPs as a high quality adjuvant for NoV VLP vaccines, but also clarified the underlying mechanism of LNPs as a potent immunostimulatory component for improving protein subunit vaccines.

**IMPORTANCE:**

With the rapid development of mRNA vaccines, recurrent studies show that lipid nanoparticles (LNPs) have adjuvant activity. However, the mechanism of its adjuvant effect in protein vaccines remains unknown. In this study, we found that the LNP-adjuvanted norovirus bivalent virus-like particle vaccines led to durable antibody responses as well as Th1-type cytokine-producing CD4^+^ T cell and CD8^+^ T cell responses, which exceeded the efficiency of the conventional adjuvant aluminum hydroxide. Mechanistically, LNPs activated innate immune responses in a type I IFN-dependent manner and were partially dependent on Toll-like receptor 9, thus affecting the adaptive immune responses of the vaccine. This work unveils that LNPs as a potent immunostimulatory component may be ideal for generating CD8^+^ T cell and B cell responses for recombinant protein vaccines.

## INTRODUCTION

Noroviruses (NoVs) are the leading pathogens causing acute gastroenteritis, affecting all age groups, with high morbidity and mortality rates among older people, immunocompromised individuals, and young children in developing countries (1). The World Health Organization highlighted NoVs as a priority for vaccine development (2). NoV virus-like particles (VLPs), which mimic the organization and conformation of native viruses but lack a viral genome, are common candidates currently undergoing clinical studies (3–6). Currently, the most advanced vaccine in development is the bivalent GI.1/GII.4 VLP vaccine adjuvanted with aluminum hydroxide (Alum) developed by Takeda Pharmaceutical Co., Ltd., and a phase II trial (NCT02153112) was conducted in infants and children (3). Moreover, more NoV VLP vaccine candidates with different valences, which are adjuvanted with Alum, are undergoing clinical (NCT04188691, NCT04941261, NCT05281094, and NCT04563533) or preclinical research (7).

Recombinant protein vaccines are generally poorly immunogenic and require the addition of an adjuvant to enhance the induction of durable protective immune responses (8). Until recently, aluminum salt was the most commonly used adjuvant in human vaccines worldwide. However, when recombinant proteins or inactivated viral vaccines are administered with aluminum adjuvant, they usually inefficiently induce the cellular immune responses, which are essential for vaccines targeting intracellular pathogens. The immunological response to norovirus infection remains incompletely understood because of the lack of cell culture systems and small animal models of human norovirus (HNoV) infection. Some studies have found that strain-specific NoV blocking antibodies were associated with protection from NoV infection in a strain-dependent manner in humans (9, 10). It was reported that both CD4^+^ and CD8^+^ T cells are required for efficient clearance of primary murine norovirus (MNoV) MNV1.CW3 infection from the intestine and intestinal lymph nodes (11). Adoptive transfer of MNoV-specific CD8^+^ T cells could significantly reduce intestinal viral load in rag1^–/–^ mice challenged by MNV1.CW3 (12). In humans, observational data from immunocompromised and immunocompetent hosts suggest a similar need for broad cellular immunity to achieve viral control (13, 14). Therefore, another challenge in developing norovirus vaccines is to induce not only an efficient humoral immune response but also a robust cellular immune response.

Lipid nanoparticles (LNPs) are delivery vehicles that greatly influence the field of vaccination (15, 16). LNPs are composed of ionizable lipids, cholesterol, lipids conjugated with polyethylene glycol, and helper phospholipids (17). Phospholipids and cholesterol have structural and stabilizing effects, whereas PEGylated lipids support prolonged circulation. Ionizing lipids combine with negatively charged mRNA molecules and enable mRNA to release from the endosome to the cytosol for translation (18). LNPs containing mRNA-encoding protein antigens have been used to induce strong immune responses, including antigen-specific antibody and T cell responses, in mice and rhesus monkeys (19–22). A recurrent finding in mouse studies on mRNA delivery is the induction of a type I interferon (I-IFN) response (23–25), which is known to support CD8^+^ T cell responses (26, 27). The BNT162b2 mRNA vaccine induces CD8^+^ T cell responses in I-IFN-dependent melanoma differentiation-associated protein 5 (MDA5) signaling (25). The influenza mRNA vaccine could induce I-IFN-polarized innate immunity, which leads to an effective vaccine-specific response in the rhesus macaque model (19). However, whether LNPs contribute to the initiation of the innate immune response has not been clearly determined.

Although LNPs have been used in various mRNA vaccine preparations for many years (28), the source of the adjuvant activity and the scope of application of such adjuvants remain unclear (29). Increasing evidence suggests that LNPs may have intrinsic adjuvant effect and could be used as adjuvants for recombinant protein vaccines. When co-delivered with protein antigens derived from dengue virus or hepatitis B virus, LNPs could enhance antigen-specific antibody, CD4^+^ T cell, and CD8^+^ T cell responses in mice (30, 31). LNPs as an adjuvant for subunit protein vaccines (dengue virus) could induce a significant immune response, which was also found in guinea pigs and non-human primates (30). A recent study showed that LNPs could promote robust induction of T follicular helper cell, B cell, and humoral responses when utilized in influenza virus or severe acute respiratory syndrome coronavirus 2 subunit protein vaccines, which are dependent on interleukin (IL)-6 production but not on myeloid differentiation primary response 88 (MyD88)- or mitochondrial antiviral signaling protein (MAVS)-dependent sensing of LNPs (32). However, the mechanism underlying the use of LNPs as a protein vaccine adjuvant remains poorly understood.

In this study, we compared the adjuvant activity of LNPs and Alum for a bivalent GI.1/GII.4 NoV VLP vaccine. The results showed that LNP-adjuvanted VLP vaccines induced earlier production of binding, blocking, and neutralizing antibodies and stronger Th1 subclass antibody IgG2a, IgG2b, and IgG3 antibody responses than Alum. Long-term immune monitoring showed that the antibody levels were comparable to those in the Alum-adjuvanted group. It is crucial that the LNP-adjuvanted VLPs could induce stronger CD4^+^ and CD8^+^ T cell immune responses than Alum. Mechanistically, LNPs activate the innate immune response depending on the presence of the ionizable lipid component in a Toll-like receptor (TLR) 9- and I-IFN-dependent manner, thereby affecting the adaptive immune response of the vaccine. This conclusion was supported by RNA-seq analysis and *in vitro* cell experiments as well as by the deeply blunted T cell responses in I-IFN receptor knockout (IFNαR1^−/−^) mice immunized with LNP-formulated VLP vaccines. This study indicated the potential of LNPs as a high-quality adjuvant for norovirus VLP vaccines and identified LNPs as a potent immunostimulatory component for improving protein subunit vaccines.

## RESULTS

### LNP-adjuvanted vaccines augment antibody responses characterized by earlier antibody production and a more balanced IgG2a/IgG1 ratio than Alum

**Fig 1:**
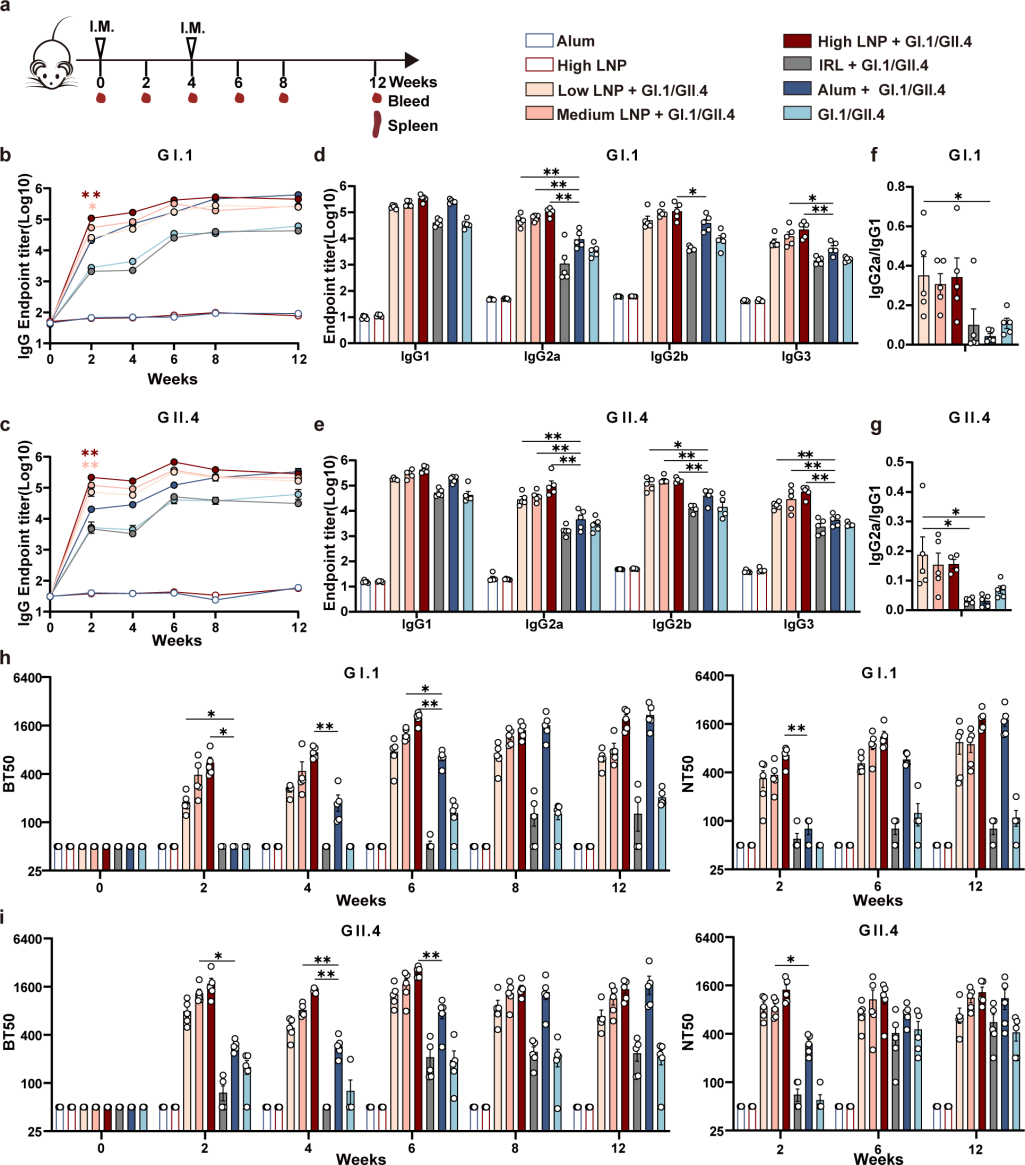
LNP-adjuvanted vaccines augment antibody production. (a) Immunization schedule. BALB/c mice (*n* = 5/group) received two shots i.m. at weeks 0 and 4; GI.1 and GII.4 VLPs were mixed with low, medium, and high doses of LNP, Alum, or IRL. Alum, high LNP, and GI.1/GII.4 VLPs were set as the control. Serum was collected every 2 weeks between week 0 and 8 and at week 12 post-priming. Binding IgG against (b) GI.1, (c) GII.4 VLPs were detected using enzyme-linked immunosorbent assay (ELISA). IgG subclasses against (d) GI.1, (e) GII.4 VLPs were detected using ELISA collected at week 6. IgG2a/IgG1 ratio was calculated for GI.1 (f) and GII.4 (g). Blockade antibody titers were determined using a VLP-mucin binding blockade assay, and neutralizing antibodies were determined using NoV VLP-HiBiT-based live-cell entry assay system. Blockade antibody results against (h) GI.1 and (i) GII.4 VLPs were shown on the left, and neutralizing antibody results were shown on the right. Data are shown as mean ± SEM. (b and c) shows statistical differences between the low, medium, and high LNP + GI.1/GII.4 group and Alum + GI.1/GII.4 group at week 2. (d–i) shows statistical differences between the low, medium, and high LNP + GI.1/GII.4 groups and the Alum + GI.1/GII.4 group. [Two-way analysis of variance (ANOVA) with Tukey’s multiple comparison test for b–e, h–i; one-way ANOVA with Tukey’s multiple comparisons test for f and g. ^*^P < 0.05；^**^P < 0.01].

It has been reported that LNPs may have intrinsic adjuvant activity (30–33). To further verify that LNPs might be repurposed as a standalone adjuvant for protein vaccine platforms, we explored the adjuvant activity of LNPs for norovirus bivalent VLP vaccines with the genotype GI.1/GII.4. VLPs were prepared as described previously (7) and detected using SDS-PAGE and transmission electron microscopy (TEM) (Fig. S1a through c). LNPs were prepared at a ratio of four lipids (Dlin-MC3-DMA:DSPC:cholesterol:DMPE-PEG2000) of 50:10:38.5:1.5. TEM and dynamic light scattering (DLS) analyses showed that the average particle size of the LNPs was 219 nm and the polydispersity index was 0.059 in phosphate-buffered saline (PBS) (Fig. S1d and e). Zeta potential measurements showed a potential of −5.94 mV in PBS (Fig. S1f). Mice were then immunized intramuscularly (i.m.) with GI.1 and GII.4 VLPs combined with low, medium, and high doses of LNP individually at weeks 0 and 4, as depicted in Fig. 1a. Ionized lipids removed from LNP (IRL) at a medium dose and 250 µg Alum at an optimized dose were selected as controls. The optimal dose of Alum adjuvant for GI.1/GII.4 VLPs was determined in a dose-dependent manner (Fig. S1g through i).

Both anti-GI.1 and GII.4 VLP IgG titers showed an LNP dose-dependent trend after the initial immunization and increased significantly at week 2 (Fig. 1b and c). IgG antibodies were significantly higher in the medium or high LNP + GI.1/GII.4 group than in the Alum + GI.1/GII.4 group at week 2 (*P* < 0.05). These differences were not significant after the booster immunization. The IRL + GI.1/GII.4 induced low levels of IgG antibody titers as GI.1/GII.4 VLP without adjuvant addition, which is consistent with previous studies showing that ionized lipids (Dlin-MC3-DMA) are a vital component of LNP adjuvant activity (32), despite the different ionizable lipids used.

Next, we assessed the ability of the antisera to block the interaction of VLPs with histo-blood group antigens (HBGAs), a binding receptor for NoVs, which pig gastric mucin-type III was used as the source of HBGAs. Blockade antibody levels are correlated with protection against clinical NoV gastroenteritis (34). Both GI.1- and GII.4-specific blocking antibodies showed an LNP dose-dependent trend after the initial immunization, which obviously increased at week 2. Specifically, compared with the Alum + GI.1/GII.4 group, the low, medium, and high LNP + G1.1/GII.4 groups showed much higher GI.1- and GII.4-specific blocking antibody titers at weeks 2, 4, and 6. However, at week 12, the blocking antibody titers were similar between high LNP + G1.1/GII.4 group and Alum + GI.1/GII.4 group. The IRL + GI.1/GII.4 group induced comparable GI.1- and GII.4-specific blocking antibody titers in the G1.1/GII.4 group (Fig. 1h and i). The results from the blocking antibodies resembled those from the binding IgG antibodies. In addition, the neutralizing ability was tested by VLP-HiBiT entry assay. Consistent with HBGA blocking results, LNP-adjuvanted groups produced earlier neutralizing antibodies at week 2. And the neutralizing antibody titer in the Alum-adjuvanted group was similar to that in the high LNP + GI.1/GII.4 group at week 12 (Fig. 1h and i).

We further detected the magnitude of the IgG subclass profile, including IgG1, IgG2a, IgG2b, and IgG3, against GI.1 or GII.4 VLPs at week 6 post-priming. The low, medium, and high LNP + GI.1/GII.4 groups induced much higher GI.1 or GII.4 VLP-specific Th1 subclass antibody IgG2a, IgG2b, and IgG3 titers, while inducing comparable Th2 subclass antibody IgG1 titers compared with the Alum + GI.1/GII.4 group (Fig. 1d and e). The IgG2a/IgG1 ratios in the LNP-adjuvanted group were much higher than those in the Alum group, indicating that the LNP adjuvant improved the Th1 subclass antibody response compared with Alum (Fig. 1f and g). To further verify this phenomenon, we detected IgG subclass dynamics. All LNP groups induced much higher Th1 subclass antibody IgG2a, IgG2b, and IgG3 titers than Alum at most time points (Fig. S2a through d). The Alum-adjuvanted group induced the highest Th2 subclass antibody IgG1 at weeks 8 and 12. The IgG2a/IgG1 dynamic ratio showed that the LNP adjuvant greatly enhanced the IgG2a/IgG1 ratio at all time points compared with the Alum-adjuvanted vaccine (Fig. S2e and f), which indicates a switch from a Th2 biased response to a more balanced Th1/Th2 response than Alum.

Because germinal centers (GCs) are essential for the development of high-quality antibody responses (35), antigen-specific GC B cells from mouse draining lymph nodes (dLNs), including the popliteal, inguinal, and iliac LNs, were detected on day 10 after immunization. The high LNP + GI.1/GII.4 group showed significantly higher total GC B cell numbers (*P* < 0.01) and GI.1 (*P* < 0.01) or GII.4 (*P* < 0.05) VLP-specific GC B cell numbers than the Alum + GI.1/GII.4 group (Fig. 3f through h). The stronger GC B cell responses may be related to the earlier humoral immune responses induced by LNPs, as shown in Fig. 1.

These experiments demonstrate that the LNP formulation used in this study has intrinsic adjuvant activity in assisting bivalent NoV VLP vaccines, which relies on ionizable lipid components. In addition, compared with the conventional Alum adjuvant, the LNP adjuvant could induce stronger and earlier antibody responses and a more balanced Th1/Th2 response than Alum. Despite early advantages in the LNP-adjuvanted group, the antibody responses in Alum-adjuvanted group was still able to “catch up” over time.

### LNP-adjuvanted vaccines induce strong NoV-specific Th1-type cytokine-expressing CD4^+^ and CD8^+^ T cell immune responses

**Fig 2:**
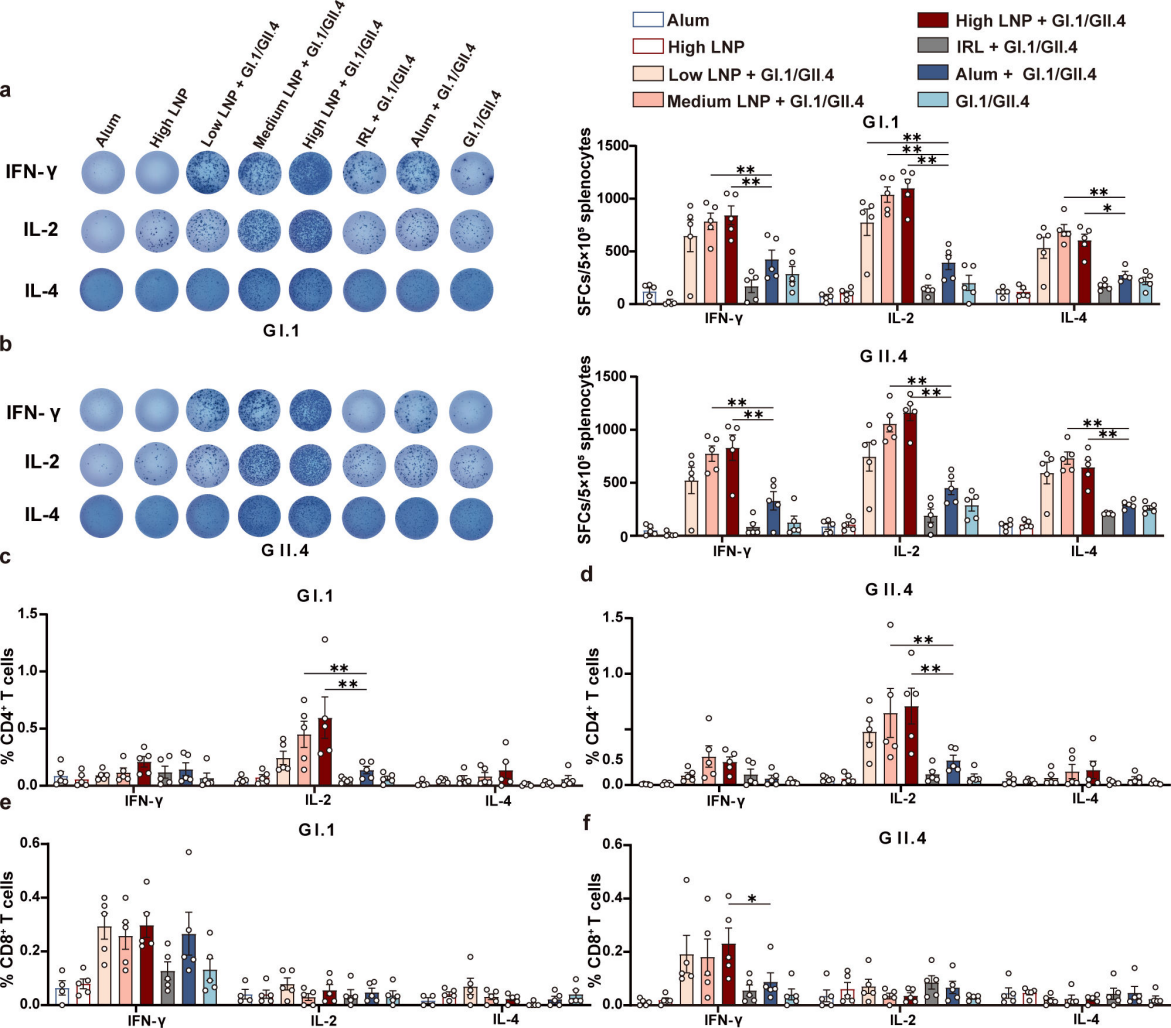
Memory T cell immune responses in vaccinated mice. (a and b) ELISpot was performed to determine the ability of splenocytes re-stimulated with GI.1 VLPs (a) or GII.4 VLPs (b) to release IFN-γ, IL-2, and IL-4 at week 12. On the left, panels display the representative ELISpot plots. On the right, panels demonstrate scatter dot plots. GI.1 VLP-specific CD4^+^ (c) and CD8^+^ (e) T cells or GII.4 VLP-specific CD4^+^ (d) and CD8^+^ (f) T cells were measured using flow cytometry at week 12. Data are shown as mean ± SEM. All data only show statistical differences between the low, medium, and high LNP + GI.1/GII.4 groups and the Alum + GI.1/GII.4 group. (Two-way analysis of variance with Tukey’s multiple comparisons test. ^*^P < 0.05；^**^P < 0.01).

To detect the memory T cell immune responses, mouse splenocytes were harvested and re-stimulated *in vitro* with GI.1 or GII.4 VLPs and then detected using enzyme-linked immunospot (ELISpot) and intracellular cytokine staining assays at week 12. It showed that the number of Th1-type cytokine-producing splenocytes, such as IFN-γ or IL-2, significantly increased in medium LNP + GI.1/GII.4 and high LNP + GI.1/GII.4 groups compared with the Alum + GI.1/GII.4 group and showed a dose effect in three LNP-adjuvanted groups (Fig. 2a and b). Splenocytes in these three LNP-adjuvanted groups also produced much higher levels of IL-4 than Alum; however, the IL-4 level was lower than that of IL-2 and IFN-γ. The ELISpot results were similar under both GI.1 or GII.4 VLP stimulation.

**Fig 3:**
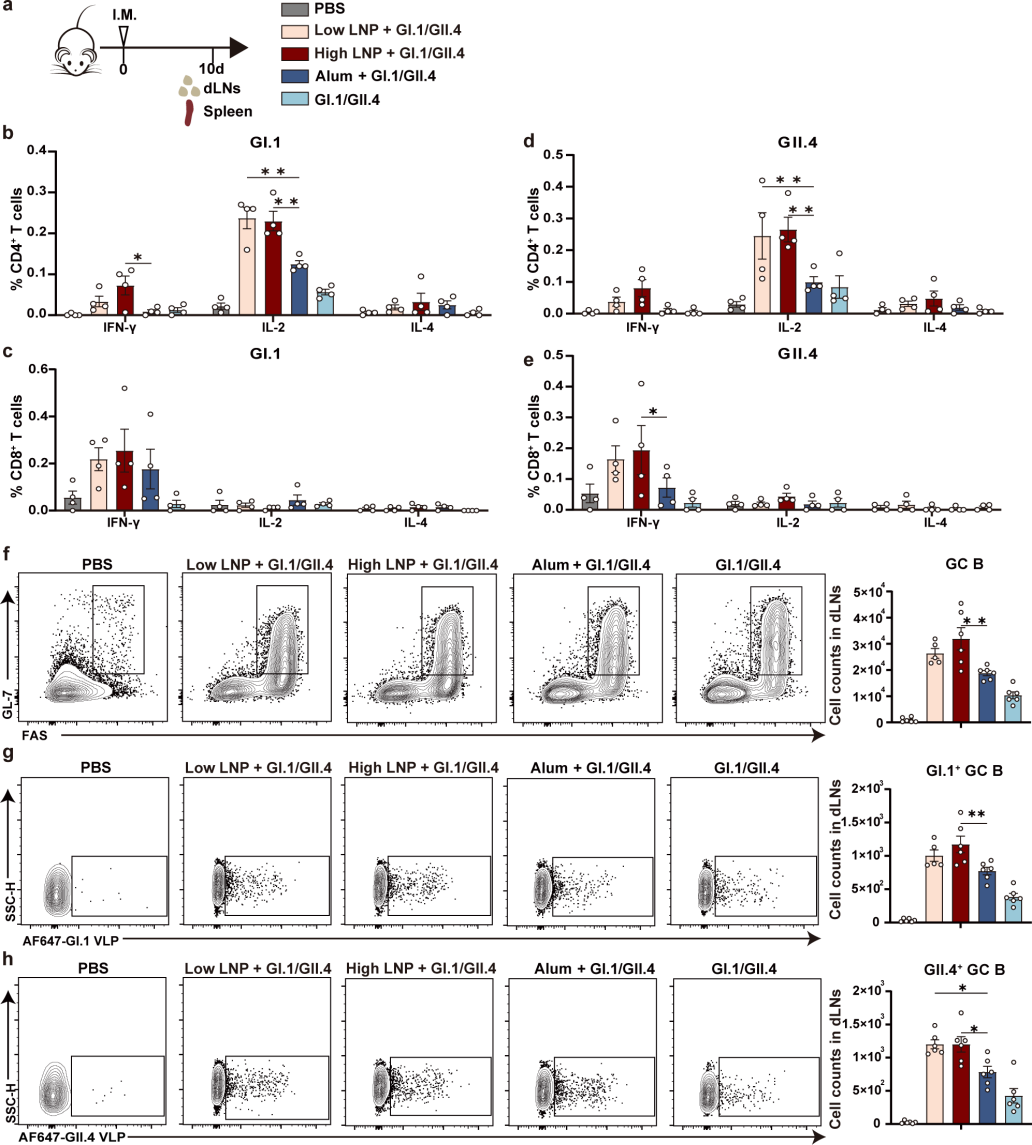
Effector T cell and GC B cell responses in vaccinated mice. (a) Immunization schedule. BALB/c mice (*n* = 6/group) received a single i.m. injection of GI.1 and GII.4 VLPs with low- or high-dose LNP or Alum. PBS and GI.1/GII.4 VLP groups were set as the control. The spleens and dLNs (popliteal, inguinal, and iliac LNs) were collected 10 days after vaccination. Intracellular cytokine staining was performed in splenocytes re-stimulated with GI.1 or GII.4 VLPs *in vitro*. Cytokines from GI.1 VLP-specific CD4^+^ (b) and CD8^+^ (c) T cells or GII.4 VLP-specific CD4^+^ (d) and CD8^+^ (e) T cells were detected (*n* = 4/group). Absolute counts of total GC B cells (f) and absolute counts of GI.1 (g) or GII.4 (h) VLP-specific GC B cells (*n* = 5-6/group) induced in dLNs. The left panels display representative images. The right panels demonstrate scatter dot plots. Data are shown as mean ± SEM. All data only show statistical differences between the low and high LNP + GI.1/GII.4 groups and the Alum + GI.1/GII.4 group. [Two-way analysis of variance (ANOVA) with Tukey’s multiple comparisons test for b–e; one-way ANOVA with Tukey’s multiple comparisons test for f–h. ^*^P < 0.05；^**^P < 0.01].

Intracellular cytokine staining assays showed that the frequencies of CD4^+^ T cells with IL-2 production were much higher in the three LNP-adjuvanted groups than that in the Alum-adjuvanted group in a dose-dependent manner. The frequencies of IFN-γ-producing CD8^+^ T cells were obviously higher in the three LNP-adjuvanted groups than that in the Alum-adjuvanted group under GII.4 stimulation; however, no discernible differences were observed under GI.1 stimulation (Fig. 2c through f). To further ascertain the cellular immune response, effector T cells were detected 10 days after a single injection (Fig. 3a through e). Consistent with the memory T cell response, the frequencies of CD4^+^ T cells with IL-2 and IFN-γ production were significantly higher in low- and high-dose LNP-adjuvanted groups than that in the Alum-adjuvanted group. The frequencies of IFN-γ-producing CD8^+^ T cells were significantly higher in high LNP + GI.1/GII.4 groups than that in the Alum + GI.1/GII.4 group under GII.4 stimulation; however, no discernible differences were observed under GI.1 stimulation.

Overall, the LNP adjuvant significantly enhanced the frequency of both Th1-type cytokine-producing T cell responses specific to GI.1/GII.4 VLP compared with Alum.

### Evaluation of gene expression and protein secretion profiles in dLNs in vaccinated mice

**Fig 4:**
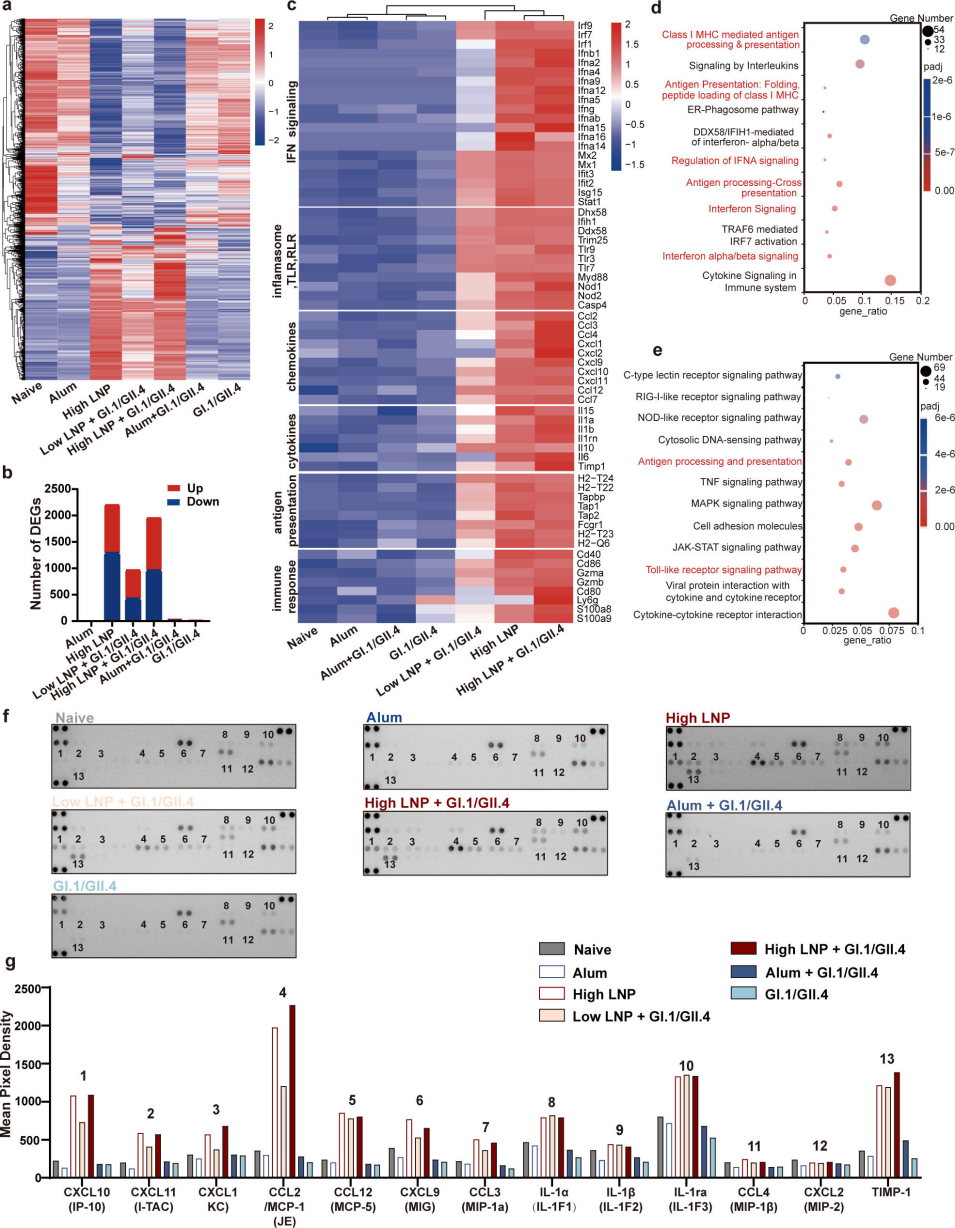
RNA-seq analysis and protein secretion profiles of dLNs in vaccinated mice. BALB/c mice (*n* = 6/group) were vaccinated i.m. with Alum, high LNP, low LNP + GI.1/GII.4, high LNP + GI.1/GII.4, Alum + GI.1/GII.4, and GI.1/GII.4. Naïve mice were set as the control. Mouse dLNs were isolated 24 h post-injection for bulk RNA-seq (*n* = 4) and mouse cytokine array (*n* = 2). (a) RNA-seq analysis of hierarchical clustering of significant differentially expressed genes in different groups. (b) DEG numbers of each group are indicated. (DEGs are based on each group compared with the naïve group, |log2 (foldchange)| ≥ 1; adjusted *P*-value ≤0.05). (c) Heatmaps of selected DEGs are classified by their immune function. (d) Reactome and (e) Kyoto Encyclopedia of Genes and Genomes enrichment of upregulated DEGs in the high LNP group. Adjusted *P*-value ≤0.05. (f) Cytokine and chemokine levels were measured in pooled dLN lysates from two mice in each group by mouse cytokine array. Experiments were duplicated twice, and representative images from one of the two experiments are shown. (g) Mean pixel density for each cytokine or chemokine was analyzed using ImageJ.

Having established the intrinsic adjuvant effect of LNPs on NoV VLP vaccines, we further explored the underlying mechanism of LNPs in initiating an innate immune response, which is required for generating an effective adaptive immune response. DLNs were collected 24 h after immunization and subjected to RNA-seq. RNA-seq revealed that a large number of DEGs compared with naïve mice were induced in the high LNP, high LNP + GI.1/GII.4, and low LNP + GI.1/GII.4 groups, while lower numbers of DEGs were induced in the Alum, Alum + GI.1/GII.4, and GI.1/GII.4 groups. Accordingly, the number and expression levels of DEGs were relatively lower in the low LNP + GI.1/GII.4 than in the high LNP and high LNP + GI.1/GII.4 groups (Fig. 4a and b).

Further analyzing the immune function-related genes in each group, we found that LNPs upregulated numerous I-IFN pathway-related genes, such as IFN-α, IFN-β, C-X-C motif chemokine ligand (CXCL) 9, CXCL10, CXCL11, and interferon regulatory factor 7 (IRF7), in a dose-dependent manner. Genes for TLRs (TLR3, TLR7, and TLR9) and RIG-I-like receptors [ddx58 (RIG-1) and ifih1 (MDA-5)] were also upregulated, indicating that LNPs may enter the cell and activate receptors in the endosomal compartment and the cytoplasm. In addition, cytokine-related genes, such as IL-1β, IL-6, IL-10, and IL-1rn, and antigen processing and presentation genes, such as histocompatibility 2, T region locus 24 (H2-T24), antigen peptide transporter 2 (TAP2), and Fc gamma receptor 1 (Fcgr1), as well as the costimulatory molecules CD80, CD86, and CD40, were upregulated obviously in the LNP-adjuvanted groups (Fig. 4c). DEGs between the high LNP and naïve groups were used to perform pathway enrichment analysis using the reactome and Kyoto Encyclopedia of Genes and Genomes (KEGG). I-IFN signaling, class I MHC-mediated antigen processing and presentation, and TLR signaling pathways were significantly enriched (Fig. 4d and e).

The relative protein levels of inflammatory cytokines or chemokines in dLNs 24 h after immunization were further analyzed using a mouse cytokine array. Chemokines and cytokines such as CXCL9, CXCL10, CXCL11, chemokine (C-C motif) ligand (CCL) 2, CCL12, CCL3, IL-1a, IL-1ra, and tissue inhibitor of metalloprotease-1 (TIMP-1) were upregulated in the high LNP, low LNP + GI.1/GII.4, and high LNP + GI.1/GII.4 groups in a dose-dependent manner compared with the naïve, Alum, Alum + GI.1/GII.4, or GI.1/GII.4 groups (Fig. 4f and g). This result was consistent with the RNA-seq results, which indicated that LNPs could induce the pro-inflammatory microenvironment and activate the innate immune response, especially through the I-IFN signaling pathway.

### LNPs activate innate immune responses through the I-IFN signaling pathway

**Fig 5:**
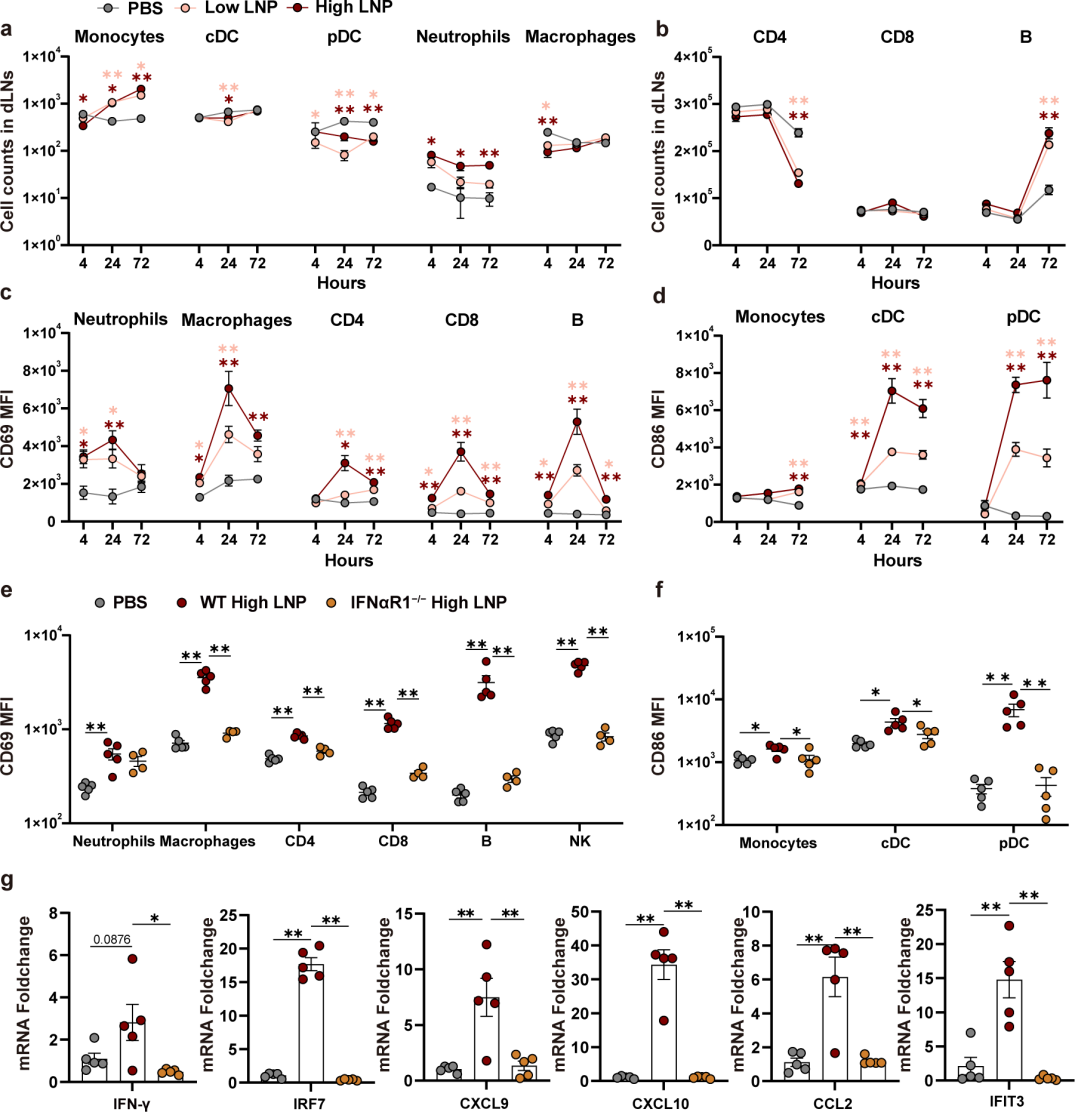
LNPs activate innate immune responses through the I-IFN signaling pathway. BALB/c mice (*n* = 5/group) were injected i.m. with low or high LNP or PBS; dLNs were isolated at 4, 24, and 72 h and analyzed using flow cytometry. (a and b) Number of each cell population in dLNs. CD69 mean fluorescence intensity (MFI) (c) and CD86 MFI (d) in activated immune cells. C57BL/6J wild-type (WT) and IFNαR1^−/−^ mice were immunized i.m. with high LNP. WT mice were injected with PBS as a control group (*n* = 5 per group). Mouse dLNs were isolated 24 h post-injection for flow cytometry analysis. (e) CD69 MFI and (f) CD86 MFI in activated immune cells. (g) qPCR was used to detect the expression levels of interferon-stimulated genes (ISGs) in dLNs of WT and IFNαR1^−/−^ mice at 24 h after injection (*n* = 5 per group). Data are shown as mean ± SEM. [Two-way analysis of variance (ANOVA) with Tukey’s multiple comparison test for a–d; one-way ANOVA with Tukey’s multiple comparison test for e–g. ^*^P < 0.05；^**^P < 0.01].

To elucidate whether the pro-inflammatory environment induced by LNPs translates into activation and changes in the composition of immune cells, we analyzed the number and activation status of leukocytes in dLNs (Fig. 5a through d). Compared with PBS, low and high LNP significantly increased the number of monocytes (CD11b^+^ Ly6C^+^) at 24–72 h, the number of neutrophils (CD11b^+^ Ly6G^+^) at 4–72 h after injection (*P* < 0.05), and the number of B cells at 72 h (*P* < 0.01). However, the numbers of cDC (CD11c^+^ MHCII^+^), pDC (CD11b^−^ CD11c^int^ PDCA-1^+^), and macrophages (CD11b^+^ F4/80^+^) changed slightly over time. Furthermore, changes in cellularity in the dLNs were accompanied by strong activation of innate immune cells, as demonstrated by CD69 expression on neutrophils, macrophages, CD4^+^ T cells, CD8^+^ T cells, and B cells, peaking at 24 h and falling at 72 h post-injection (Fig. 5c). Although the number of cDC and pDC changed slightly and even decreased at 24 h, vigorous activation of these two cell types was observed by CD86 expression at 24 h and was sustained until 72 h, indicating their vital role in initiating the immune response by LNPs (Fig. 5d).

Because the I-IFN signaling pathway was significantly enriched by RNA-seq, its impact on LNP-activated innate immune responses was further explored. Twenty-four hours after injection with high LNP, the innate immune responses were detected in lymphocytes from dLNs in wild-type (WT) and IFNαR1^−/−^ mice by flow cytometry. The data showed that, compared with WT mice, the activation of innate immune cells, measured by CD69 or CD86 expression, was significantly downregulated in many cell types, including macrophages, CD4^+^ T cells, CD8^+^ T cells, B cells, NK cells, monocytes, cDCs, and pDCs (Fig. 5e and f). Moreover, I-IFN-related or interferon-stimulated genes (ISGs) in LNs were detected using qPCR. The data showed the expression of IRF7, CXCL9, CXCL10, IFN-γ, CCL2, and IFIT3 were significantly downregulated in IFNαR1^−/−^ mice at 24 h (Fig. 5g). These results indicate that i.m. injection of the LNP adjuvant induces an extensive but transient local immunostimulatory microenvironment and that the I-IFN signal is vital for the activation of the innate immune response by LNPs.

### LNPs activate the I-IFN signaling pathway through TLR9

**Fig 6:**
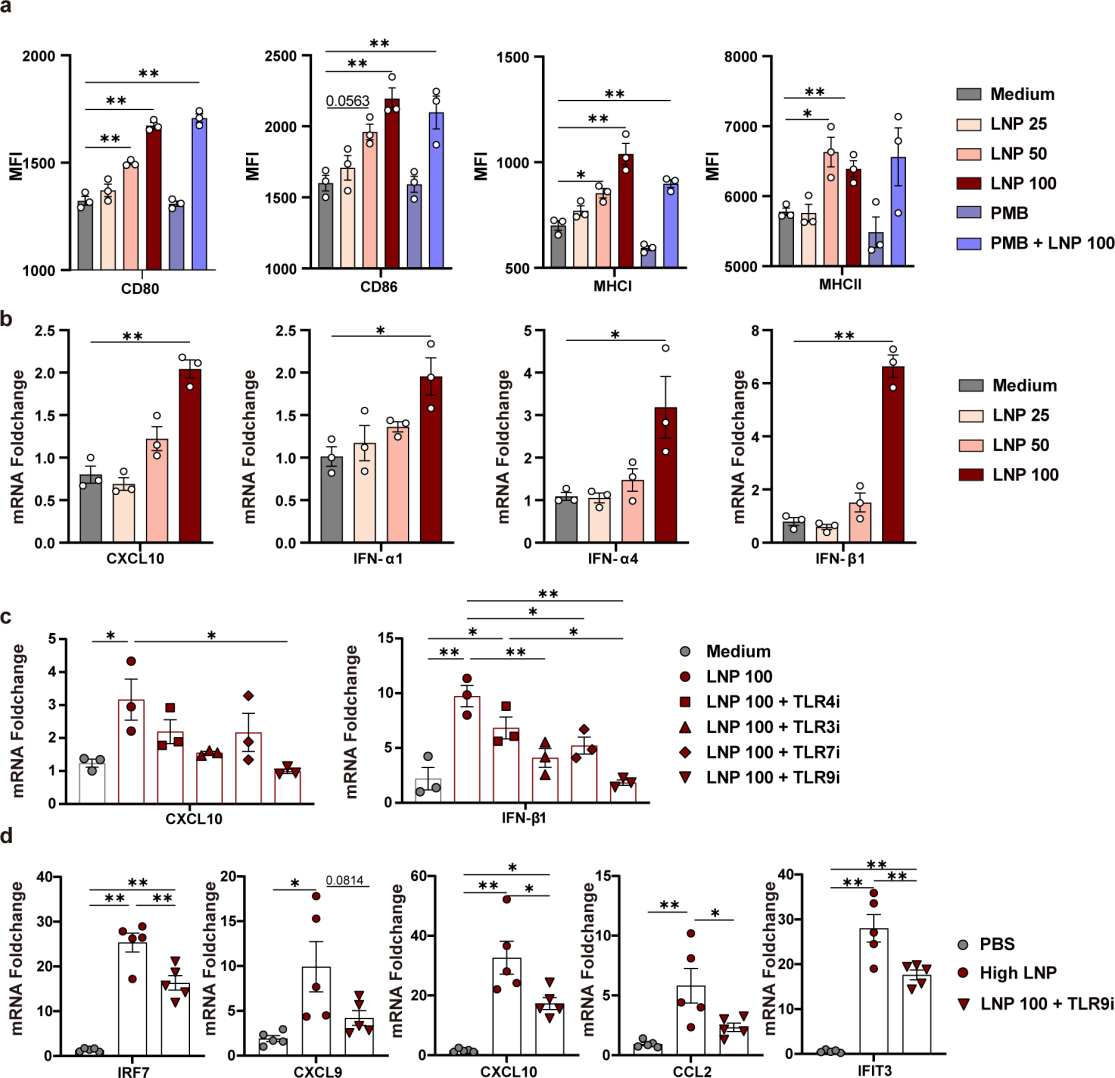
LNPs activate the I-IFN signaling pathway through TLR9. (a) BMDCs were stimulated with 25, 50, and 100 µg/mL LNP, 10 µg/mL PMB, and 10 µg/mL PMB + 100 µg/mL LNP for 24 h. BMDC activation markers CD80, CD86, MHC I, and MHC II were detected using flow cytometry. (b) BMDCs were stimulated with 25, 50, and 100 µg/mL LNP for 8 h, and the cytokines CXCL10, IFN-α1, IFN-α4, and IFN-β1 were detected using qPCR. (c) BMDCs were pretreated with TLR4, TLR3, TLR7, and TLR9 inhibitors for 1 h, and expression levels of CXCL10 and IFN-β1 were detected using qPCR in BMDCs after incubation with 100 µg/mL LNP for 8 h. (d) Expression levels of IRF7, CXCL9, CXCL10, CCL2, and IFIT3 were detected using qPCR in mouse dLNs at 24 h after i.m. injection with high LNP or high LNP with TLR9 inhibitor (50 µg per mouse). (*n* = 5 per group, C57BL/6J). (a and b) shows statistical differences between each group and the medium control group (one-way analysis of variance with Tukey’s multiple comparison test. ^*^P < 0.05; ^**^P < 0.01).

Because LNPs induced vigorous and sustained dendritic cell (DC) activation *in vivo* in mice, we further tested the effect of LNPs on Bone marrow-derived dendritic cell (BMDC) activation *in vitro*. BMDCs were obtained by culturing isolated mouse bone marrow cells in the presence of granulocyte-macrophage colony-stimulating factor (GM-CSF), and the purity of the BMDCs reached >90% using flow cytometry (Fig. S3d). LNPs significantly upregulated the expression of costimulatory molecules such as CD80, CD86, MHC I (H-2Kb/H-2Db), and MHC II (I-A/I-E) in BMDCs in a dose-dependent manner (Fig. 6a), indicating BMDC maturation and activation. To further exclude the effect of endotoxin contamination, BMDCs were stimulated with polymyxin B (PMB) or PMB with LNPs for 24 h. PMB, which blocks the biological effects of lipopolysaccharide by binding to lipid A, did not block the activation of BMDCs by LNPs (Fig. 6a). This confirmed that LNP-activating BMDCs were not due to lipopolysaccharide contamination.

Since RNA-seq showed that LNPs induced extensive upregulation of I-IFN-related genes, the expression of these genes in BMDCs stimulated with LNPs for 8 h was detected using qPCR. The results showed that LNPs significantly induced the expression of IFN-α1, IFN-α4, IFN-β1, and CXCL10 in a dose-dependent manner (Fig. 6b). Therefore, LNPs can induce the I-IFN signaling pathway in BMDCs.

Since TLR9, TLR7, and TLR3 were significantly upregulated by LNPs and have been reported to be closely related to I-IFN signaling, we further determined whether LNP-induced BMDC activation depends on the TLR signaling pathway. BMDCs were pretreated with small-molecule inhibitors of TLR4 (TLR4i, TAK-242), TLR3 (TLR3i, CU CPT4a), TLR7 (TLR7i, eupatorine), or TLR9 (TLR9i, ODN2088) separately, followed by the addition of LNP for 8 h. Then, the expression of IFN-β1 and CXCL10 was analyzed using qPCR (Fig. 6c). It showed that TLR9 inhibitor significantly reduced CXCL10 (*P* < 0.05) and IFN-β1 (*P* < 0.01) expression in LNP100-treated BMDCs and that TLR3 and TLR7 inhibitors partially reduced IFN-β1 and CXCL10 expression in LNP100-treated BMDCs, with statistical significance just for IFN-β1. However, the TLR4 inhibitor only partially inhibited the production of both cytokines, with no statistical significance. Consistent with the effects of TLR9 inhibitors *in vitro*, mice injected with TLR9 inhibitors significantly inhibited LNP-stimulated I-IFN signaling-related genes, IRF7, CXCL9, CXCL10, CCL2, and IFIT3 (Fig. 6d).

In summary, LNPs activate I-IFN signaling partially by activating the TLR9 pathway, thereby promoting DC activation for efficient antigen presentation.

### IFNαR1 knockout impacts LNP-mediated cellular and humoral immune responses in mice

**Fig 7:**
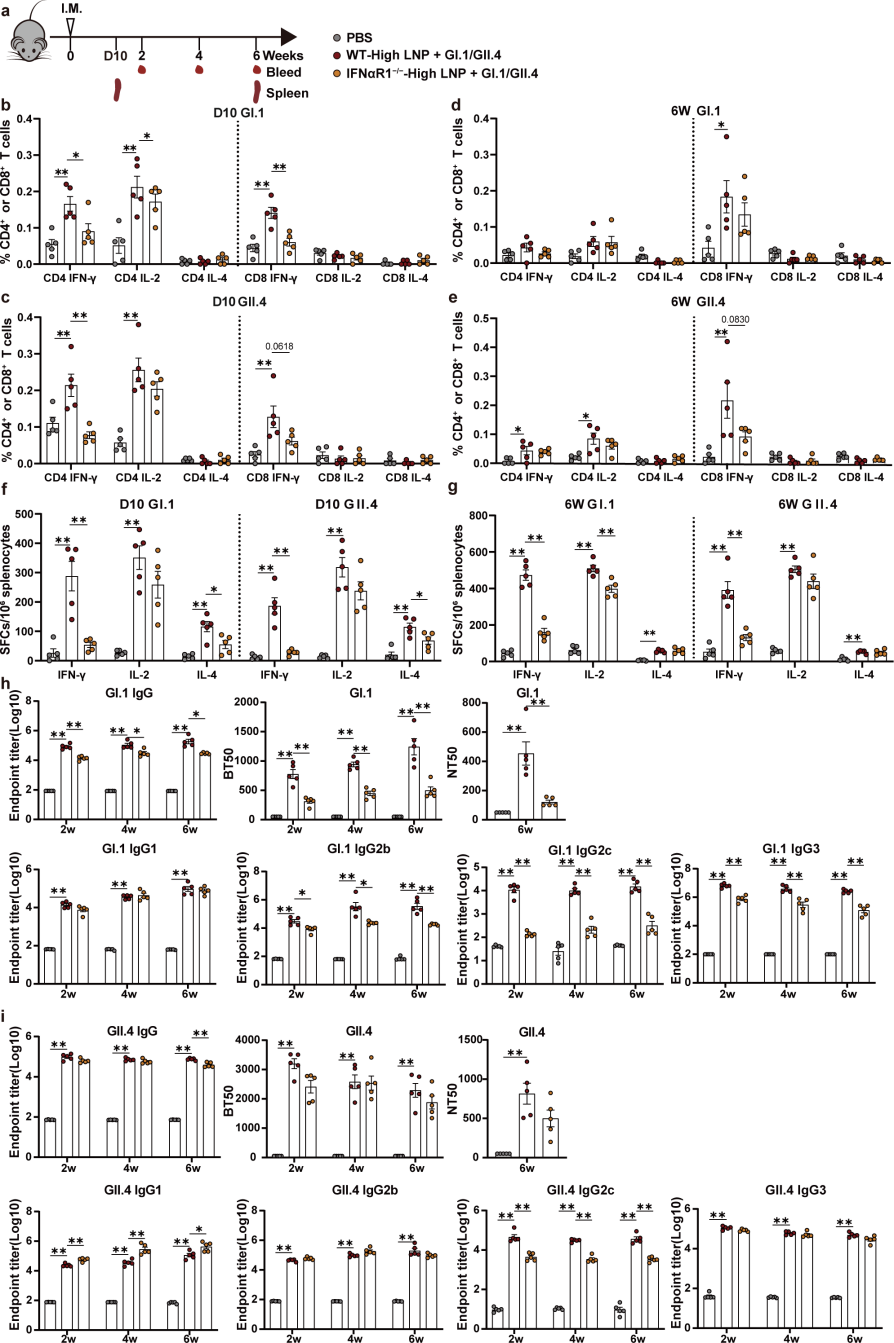
IFNαR1 knockout impacts LNP-mediated cellular and humoral immune responses in mice. (**a**) Immunization schedule. C57BL/6J WT and IFNαR1^−/−^ mice were injected i.m. with high LNP + GI.1/GII.4 VLP, and WT mice were inoculated with PBS as a control group (*n* = 10 per group). Then, GI.1 or GII.4 VLP-specific CD4^+^ and CD8^+^ T cells producing IFN-γ, IL-2, and IL-4 were measured using flow cytometry at day 10 (b–c) or at week 6 (d–e). ELISpot was used to detect the ability of splenocytes to release IFN-γ, IL-2, and IL-4 after restimulation with GI.1 or GII.4 VLP on day 10 (**f**) or at week 6 (**g**). GI.1 VLP (**h**) and GII.4 VLP (**i**) specific-binding antibodies IgG, blocking antibodies, neutralizing antibodies determined using NoV VLP-HiBiT-based live-cell entry assay system, and IgG subclasses were detected. Data are shown as mean ± SEM. All data show the statistical analysis of each group compared with WT-high LNP + GI.1/GII.4. [One-way analysis of variance (ANOVA) and Tukey’s multiple comparisons test for b–g and NT50 in h and i. Two-way ANOVA test with Tukey’s multiple comparisons test for the other data in h and i. ^*^P < 0.05；^**^P < 0.01].

I-IFN is a cytokine family with broad functions, including the activation of the innate immune response and the shaping of the adaptive immune response. I-IFN can aid in the activation of DCs and lead to the activation and type 1 immune polarization of T cells (26). Therefore, we explored the effect of I-IFN signaling on the adaptive immune response induced by LNP-adjuvanted NoV VLP vaccines. WT or IFNαR1^−/−^ mice were i.m. injected with high LNP + GI.1/GII.4 VLP. Effector T cells on day 10 and memory T cells at week 6 from mouse spleens were detected using flow cytometry or ELISpot after restimulation with GI.1 or GII.4 VLPs *in vitro* (Fig. 7a).

The flow cytometry results showed that on day 10, IFN-γ-producing CD4^+^ and CD8^+^ T cell ratios were significantly lower in IFNαR1^−/−^ mice compared with those in WT mice. There is a significant decrease in IL-2-producing CD4^+^ T cell ratios under GI.1 VLPs stimulation (*P* < 0.05), and there was no statistical significance for decrease in IL-2-producing CD4^+^ T cell ratios under GII.4 VLPs stimulation(Fig. 7b and c). At week 6, memory T cells showed a similar decreasing trend in IFNαR1^−/−^ mice compared with WT mice; however, there was no statistical significance (Fig. 7d and e).

The results from the ELISpot assay showed that a significantly lower level of IFN-γ was produced by splenocytes from IFNαR1^−/−^ mice compared with that from WT mice on day 10 or at week 6 after immunization (Fig. 7f and g). Similar results were observed for IL-2 and IL-4 on day 10 and week 6, although the differences were seldom statistically significant.

In addition, GI.1 and GII.4 VLP-specific IgG, IgG1, IgG2b, IgG2c, and IgG3 and blocking antibody and neutralizing antibodies were detected. Compared with WT mice, GI.1 VLP-specific IgG, IgG2b, IgG2c, and IgG3 and blocking and neutralizing antibodies were significantly reduced in IFNαR1^−/−^ mice (Fig. 7h) and GII.4 VLP-specific IgG, IgG2c, and IgG3 and blocking and neutralizing antibodies showed similar decreased trends in IFNαR1^−/−^ mice, with statistically significant results just for IgG2c. In addition, Th2 subclass IgG1 antibody titers were not obviously affected specific for GI.1 VLPs and even significantly higher specific for GII.4 VLPs in IFNαR1^−/−^ mice than WT mice (Fig. 7i). In contrast, the immune response of Alum-adjuvanted VLPs were not apparently affected in IFNαR1^−/−^ mice compared to those in WT mice as shown in Fig. S4.

These data show that the LNP-mediated I-IFN signaling pathway is critical for the induction of Th1-type cytokine-expressing CD4^+^ and CD8^+^ T cell immune responses as well as for blocking antibody and Th1 subclass antibody production.

## DISCUSSION

In this study, novel adjuvant LNPs were used to improve the immunogenicity of a bivalent NoV VLP vaccine, which was manifested by the rapid production of antibodies and Th1-type cytokine-producing CD4^+^ and CD8^+^ T cell immune responses. Compared with the traditional Alum adjuvant, we found that LNP adjuvant-induced antibody levels peaked in the 6th week and then began to decline slightly, as shown in Fig. 1. Owing to its well-known sustained-release effects, Alum increased antibody levels over time to levels similar to those of the LNP-adjuvanted group. The Alum adjuvant adsorbs and granularizes soluble antigens that are retained at the site of injection, thus allowing time to recruit antigen-presenting cells by releasing cytokines and inducing a local inflammatory response (36, 37). LNPs, as adjuvants for protein vaccines, rapidly activate the immune response but induce a less sustained humoral immune response than Alum, which may be improved through modification of LNPs with sustained release characteristic in the future.

In our study, we compare the antibody productions, HBGA blocking activities, and T cell responses to demonstrate the superiority of LNP over Alum as adjuvant for NoV VLP vaccines. Whether these immune responses correlated to protection against virus challenge needs further investigation. So far, the HBGA blocking assay has been used extensively to assess the protective potential of candidate NoV vaccine-elicited antibodies. The availability of challenge model in mice and neutralizing model in cells by real viruses still carries important limitations. In view of the differences in receptors (38) and T cell epitopes between HNoV and MNoV (39), MNoV challenge may not accurately evaluate the efficacy of HNoV vaccines. Cultivation of NoVs in human intestinal enteroids (HIEs) makes it possible to evaluate virus neutralization (40). However, the limited supply and the required high-level handling skills for the HIE system restrict its application. To test the efficacy of neutralizing activity, the NoV VLP-HiBiT-based live-cell entry assay system was applied in our study (41). Compared to free HBGAs, cell membrane-associated HBGAs present more authentic orientations and interactions with other membrane lipids and proteins. This system is performed by live cells, making it possible to monitor not only the attachment but also the internalization/endocytosis steps in the viral entry process.

The results from RNA-seq and mouse cytokine array showed that intramuscular injection of LNPs upregulated I-IFN genes and I-IFN-related genes (IFN-α, IFN-β, MX-1, IFIT3, ISG15, and IRF7) as well as chemokines (CXCL9, CXCL10, CXCL11, CCL2, and CCL3), indicating that LNPs significantly induced I-IFN signaling. Accumulating data suggest that I-IFN enhances T cell immunity by acting indirectly or directly on T cells. I-IFN indirectly affects T cell priming through the upregulation of costimulatory molecules on APC and acts directly as an activating stimulator to prevent failure of the T cell response (42). In addition, I-IFN also exhibits a direct stimulatory effect on immune cells by promoting IFN-γ secretion (43). Previous studies showed that DC-specific IFNαR^−/−^mice are unable to reject highly immunogenic tumor cells due to defects in antigen cross-presentation to CD8^+^ T cells (44). This evidence strongly suggests that I-IFN acts through DC to promote T cell immunity. Our work highlights that I-IFN promotes the induction of CD8^+^ T cell responses, although I-IFN may not function alone but rather function in combination with other cellular factors, such as I-IFN induction of the ISGs CXCL9, CXCL10, and CXCL11,which are important for the recruitment of CD8^+^ cells (45). In summary, we provide strong evidence that I-IFN plays an indispensable role either directly or indirectly in LNP-adjuvanted vaccines.

However, the molecular mechanism by which I-IFN promotes B cell and antibody responses remains unknown. Innate immune response detection showed that IFNαR knockout affects the expression of CD69, an early leukocyte activation marker, or the costimulatory molecule CD86 in various cell types, which may explain how I-IFN signal promotes humoral immunity, as observed in a previous study (46). Moreover, RNA-seq analysis showed IL-6 upregulation, which may be one of the reasons for antigen-specific GC B amplification. IL-6 is a cytokine critical for early T follicular helper cell differentiation in mice (47), which can explain why the LNP adjuvant improved the B cell immune response. In addition to antibody production, B cells are efficient antigen-presenting cells and express a variety of pathogen recognition receptors including TLR9 in mice or humans (48, 49). It was reported that mouse naïve B cells can respond to CpG ODN through TLR9 activation, even in the absence of BCR triggering or T cell help (50). The proliferation and differentiation of human naïve B cells required a combination of three signals, BCR triggering, cognate T cell help, and TLR stimulation (51). In our study, we found that LNP could activate DCs through TLR9. Meanwhile, we observed that B cells in lymph nodes could also be significantly activated by LNP after 24-h injection, and vigorous GCB responses were induced in LNP-adjuvanted group at day 10. The mechanism by which LNP directly affects B cells and its possible impact on later antibody production need to be further explored.

In our study, serum antibodies IgG, IgG1, IgG2a/2c (named IgG2c in C57BL/6 mice and hereinafter referred to as IgG2a/c), IgG2b, and IgG3 were analyzed. In contrast to serum antibodies, fecal IgA levels were very low to detect (data not shown). This may be due to the route of immunization, since i.m. immunization usually induces system immune response instead of mucosal response. According to our previous report (52), when administered via intranasal (i.n.) + i.n. or i.m. + i.n. regimen, a quadrivalent NoV vaccine based on a chimpanzee adenovirus vector induced high IgA antibody titers (2^6^ to 2^8^) in bronchoalveolar lavage fluid and saliva in mice. It was reported that switching of antibody to the IgG2a/c isotype mediates clearance of virus and protection against lethal influenza infection (53, 54). Serum IgG2a/2c levels were significantly higher in LNP-adjuvanted group than Alum-adjuvanted group. In IFNαR1^−/−^ mice, IgG2c responses were significantly inhibited while IgG1 antibody titers were not obviously affected specific for GI.1 VLPs and even significantly higher specific for GII.4 VLPs than those in WT mice. The transcription factor T-bet induces class switch recombination to IgG2a/c (55). Type I IFNs could induce T-bet expression and support IgG2c^+^ GC B cell development (56). It was reported that Tbx21^−/−^ mice showed increased IgG1 and decreased IgG2a/c levels over the course of influenza infection, which indicated that T-bet may act both intrinsically in GC B cells to promote IgG2a/c and indirectly, presumably through TFH cytokine production, to lower IgG1 production (57). Similar results were observed in IFNar1^−/−^ mice, anti-flagellin IgG2c responses were significantly inhibited, while anti-flagellin IgG1 responses were normal and even seemed to be slightly higher compared to WT mice after boost immunization with flagellin (58). However, the molecular mechanism underlying the LNP-mediated regulation of class switching in NoV VLP immunization needs further exploration.

Effective activation of antigen-presenting cells by vaccines is a mechanism for the rapid and effective generation of vaccine-specific responses. Using multicolor flow cytometry to detect changes in cellularity in dLNs, we found that the main cell populations mobilized after LNP injection were monocytes, neutrophils, and B cells, which caused the activation of CD69 or CD86 expression in extensive immune cells. This is similar to the cell population mobilized in mice after intramuscular injection of an mRNA vaccine (25, 59). The upregulation of CCL2, CCL3, and CCL4 was consistent with that observed in the recruited monocytes. CXCL1 upregulation correlates with neutrophil recruitment (60). Furthermore, consistent with previous studies (61), this study showed that LNP upregulated the inflammatory cytokine IL-1α and IL-1β, and, more importantly, upregulated the inhibitory inflammatory cytokine IL-1ra, thus protecting them from uncontrolled systemic inflammation. Type 1 conventional DCs are specially equipped to take inert antigens, such as those found during killed or subunit vaccination, and cross-present them to activate CD8^+^ T cells (62). However, whether LNP injection may induce the activation of antigen cross-presentation, presenting a type 1 conventional DC response, thereby contributing to the antigen-specific CD8^+^ T cell response, needs to be further explored.

To explore how DCs sense LNPs, we tested the effect of TLR4, 3, 7, and 9 inhibitors and found that the TLR9 inhibitor (ODN2088) could completely inhibit CXCL10 and IFN-β1 expression and partially inhibit the transcription of ISGs *in vivo*. ODN2088 impedes TLR9 and, to some extent, TLR7 and TLR3 (63). Therefore, the inhibitory effect of ODN2088 may be mainly attributed to the inhibitory effect of TLR9, and also partly due to the inhibitory effect of TLR7 and TLR3. The exact role of TLR9 in LNP-activated immunostimulatory effect needs to be further verified in TLR9 knockout mice in the future. Some reports have indicated that cationic lipids and nanoparticles can activate TLR2, TLR4, or inflammasome pathways and induce the production of cytokines by antigen-presenting cells (61, 64–66). However, the formulation of LNPs used thus far varies; therefore, the adjuvant mechanism of LNPs may differ. RNA-seq showed that TLR3, 7, and 9, as well as RIG-I (ddx58), MDA-5 (ifih1), and inflammasome components were upregulated in mouse dLNs, indicating that the LNP adjuvant can activate other innate signaling pathways. Previous studies have shown that LNPs can activate the inflammasome NLRP3 to induce the production of IL-1β (61). In addition, nanoparticles smaller than 200 nm are believed to directly reach lymphatic drainage by advection (67), while particles between 200 and 500 nm may require DCs to circulate into the lymph. This study used LNPs based on a modified ethanol injection procedure; their size of approximately 200 nm may affect the kinetics of entering cells, but it does not affect the adjuvant activity (67, 68). Some studies found that mice require an average particle size of approximately 100 nm to generate consistently high antibody titers for mRNA vaccine; however, the optimal mRNA vaccine particle size determined in rodents may not translate to primates (67).

In summary, LNPs exhibited potent adjuvant activity for bivalent NoV VLP vaccines. The LNP adjuvant initiates the innate immune response by activating the I-IFN response in the dLNs, ultimately leading to a more effective adaptive immune response.

## MATERIALS AND METHODS

### Mice

Female BALB/c and C57BL/6J mice (6–8 weeks old) were purchased from Beijing Vital River Laboratory Animal Technology Co., Ltd. IFNαR1 knockout mice were purchased from Shanghai Model Organisms Center, Inc.

### Preparation of VLPs and LNPs

GI.1 VLPs (GenBank ID: NP_056821.2) and GII.4 VLPs (GenBank ID: KC631827.1) were produced as described in a previous study (7). Briefly, GI.1 and GII.4 VLP expression strains were induced to express VLP proteins according to the *Pichia pastoris* Expression Manual (Invitrogen). Yeast cells, after induced expression, were collected and resuspended in 0.15 M PBS containing 1 mM phenylmethylsulfonyl fluoride, homogenized at 4°C with 1,500 bar, and then the supernatant was collected after centrifugation at 12,000 rpm for 30 min. Then, 7% PEG6000 and 0.2 M NaCl were added to concentrate the supernatant. And the pellet was resuspended in 0.15 M PBS and dissolved overnight at 4°C. The next day, after centrifugation at 12,000 rpm for 30 min, the supernatant was retained and subjected to sucrose cushion and sucrose gradient ultracentrifugation as previously described (7). Fractions containing the VLPs were collected and diluted with PBS. The concentrated VLPs were quantified using SDS-PAGE and Pierce BCA Protein Assay Kit (Thermo Fisher Scientific, USA).

The LNPs were prepared using a method described before (69, 70). Briefly, lipids were dissolved in ethanol and mixed at a molar ratio of 50:10:38.5:1.5 (Dlin-MC3-DMA:DSPC:cholesterol:DMPE-PEG2000). The lipid mixture was mixed with 50 mM citrate buffer (pH 4.0) at a ratio of 1:3, then dialyzed with PBS and concentrated by 10 kd Amicon ultracentrifuge filter (EMD Millipore, Billerica, MA). The concentrated preparation was passed through a 0.45 µm filter and stored at 4°C until use. And the particle size was detected by DLS using a Zetasizer Nano ZS90 (Malvern Instruments) at room temperature. Zeta potential measurements were conducted on the same instrument as DLS.

### TEM

Electron microscopy was performed as described previously (7). VLPs and LNPs were negatively stained with 2% phosphotungstic acid, and TEM was performed using a Hitachi HT7700 transmission electron microscope (Hitachi, Japan) operated at 80 kV to analyze the shape and integrity of the VLPs and LNPs.

### Mouse immunization

The different immunization regimens were presented as follows.

#### LNP and Alum adjuvant study

Female BABL/c mice were randomly divided into eight groups (*n* = 5 per group) as follows: (i) Alum, (ii) high LNP, (iii) low LNP + GI.1/GII.4, (iv) medium LNP + GI.1/GII.4, (v) high LNP + GI.1/GII.4, (vi) IRL + GI.1/GII.4, (vii) Alum + GI.1/GII.4, and (viii) GI.1/GII.4. Mice were injected i.m. in both hind limbs at weeks 0 and 4, with a volume of 50 µL each. Blood samples were collected at 0, 2, 4, 6, 8, and 12 weeks post-priming, and sera were isolated and stored at −80°C until use. At week 12, the mice were sacrificed under anesthesia, and spleens were harvested for splenocyte preparation to detect the T cell immune response.

Female BABL/c mice were randomly divided into five groups (*n* = 5 per group) as follows: (i) PBS, (ii) low LNP + GI.1/GII.4, (iii) high LNP + GI.1/GII.4, (iv) Alum + GI.1/GII.4, and (v) GI.1/GII.4. Mice were injected i.m. in both hind limbs at week 0. Ten days after immunization, the spleens were harvested as described above to detect T cell immune responses, and dLNs, including popliteal, inguinal, and iliac LNs, were collected to detect GC B cell responses.

For animal immunization experiments in this study, 5 µg of GI.1 or GII.4 VLPs was used. In addition, 250 µg of Alum was used except in the Alum dose optimization experiment. Also, 125, 500, and 1,000 µg of LNPs represent low LNP, medium LNP, and high LNP, respectively. IRL represents LNP that removes ionizable lipid components, and the dose of 500 µg for IRL was used.

#### Alum dose optimization experiments

Female BABL/c mice were randomly divided into three groups (*n* = 5 per group) as follows: (1) 100 µg Alum + GI.1/GII.4 (2), 250 µg Alum + GI.1/GII.4, and (3) 500 µg Alum + GI.1/GII.4. The mice were immunized i.m. in both hind limbs at weeks 0 and 4. Mouse serum was collected for antibody detection at 0, 2, 4, 6, 8, and 12 weeks post-immunization.

#### Detection of innate immune response experiments

Female BABL/c mice were randomly divided into seven groups (*n* = 6 per group) as follows: (i) naïve, (ii) Alum, (iii) high LNP, (iv) low LNP + GI.1/GII.4, (v) high LNP + GI.1/GII.4, (vi) Alum + GI.1/GII.4, and (vii) GI.1/GII.4. Mice were immunized i.m. in both hind limbs. After 24 h, the mouse dLNs were collected for cytokine array detection (*n* = 2 per group) and RNA-seq (*n* = 4 per group).

Female BABL/c mice were randomly divided into three groups and were i.m. injected with PBS, low LNP, and high LNP. Mouse dLNs were obtained at 4, 24, and 72 h for detection of the innate immune response (*n* = 5 per group).

#### Detection of immune responses in IFNαR1^−/−^ mice

C57BL/6J WT mice were i.m. inoculated with PBS and high LNP, and IFNαR1^−/−^ mice were inoculated with high LNP (*n* = 5 per group). Mice were sex- and age-matched. Immune cells from the dLNs were collected for innate immune responses and cytokine detection 24 h after injection.

C57BL/6J WT mice were i.m. inoculated with PBS, high LNP + GI.1/GII.4 VLP, and IFNαR1^−/−^ mice were inoculated with high LNP + GI.1/GII.4 VLP (*n* = 10/group). Mice were sex- and age-matched. Splenocytes were collected to detect T cell immune responses on day 10 (*n* = 5 per group) and week 6 (*n* = 5 per group) post-immunization. Mouse serum was collected for antibody detection at 0, 2, 4, and 6 weeks post-immunization.

#### *In vivo* inhibition test of ODN2088

PBS, high LNP, and high LNP + ODN2088 were injected i.m. in both hind limbs of C57BL/6J mice. The dose of ODN2088 was used at 50 µg/mouse. Then, 24 h after injection, the mouse dLNs were used for cytokine detection.

### Enzyme-linked immunosorbent assay (ELISA)

VLP-specific total IgG and IgG1/IgG2a/IgG2b/IgG3/IgG2c subclass antibodies were detected using ELISA, as described in detail elsewhere (7). Briefly, sera were diluted twofold at an initial dilution of 1:500 and incubated with NoV VLP-coated plates. The plates were then incubated with a 1:50,000 dilution of horseradish peroxidase (HRP)-conjugated anti-mouse IgG (Abcam, UK) or a 1:5,000 dilution of HRP-conjugated anti-mouse IgG1/IgG2a/IgG2b/IgG3/IgG2c antibody (Southern Biotech, USA). After washing, 3,3’,5,5’-tetramethylbenzidine (TMB) substrate (NCM Biotech, China) was added and then were stopped by adding 2 M sulphuric acid. Optical density was measured at 450 nm using a microplate spectrophotometer (Agilent, USA). The average endpoint titer for each serum sample was determined as the reciprocal of the highest serum dilution with an optical density above the set cutoff value.

### VLP-mucin binding blocking assay

The ability of antisera to block the interaction of VLPs with HBGAs was evaluated using a blocking assay, as described in detail elsewhere (7). Briefly, 10 µg/mL pig gastric mucin-type III was coated on an ELISA plate. Individual sera were subjected to twofold gradient dilutions with an initial 100-fold dilution, and equal volumes of mixtures of NoV VLPs and serially diluted serum samples were incubated at room temperature for 1 h and 15 min before being added to the plates. Bound VLPs were detected using NoV VLP type-specific antibodies, followed by incubation with HRP-conjugated goat anti-rabbit IgG (Abcam, UK) and TMB substrate. The OD450 was measured as described above. Maximum binding was determined using VLPs without mouse serum. The blocking titer 50 (BT50) was expressed as the reciprocal of the highest serum dilution that blocked 50% of the maximal VLP binding. An arbitrary titer (BT50 = 50) was assigned to samples with a blocking index of <50% at the lowest serum dilution of 1:100.

### Neutralizing antibody detection using NanoLuc binary technology

NoV VLP-HiBiT-based live-cell entry assay system (a gift from professor Huang and Zhang) was adopted to detect the neutralizing antibodies. According to the previous report (41), 293T-FUT2-LgBiT cells were cultured in poly-L-Lys-coated 96-well plates at a concentration of 4 × 10^4^ cells per well in Dulbecco’s modified Eagle medium (DMEM) supplemented with 10% fetal bovine serum (FBS), sodium pyruvate, 100 U/mL penicillin, and 100 µg/mL streptomycin at 37°C for 24 h. Then, the cells were chilled at 4°C for 1 h. Meanwhile, an equal-volume mixture of VLP-HiBiT with 1.5 µg/mL and serially diluted serum samples were incubated for 1 h at room temperature and then chilled at 4°C for 10 min. After removal of the culture supernatant, cooled VLP/serum mixtures were added to prechilled 293T-FUT2-LgBiT cells and incubated at 4°C for 1 h. After washing with cold PBS, Nano-Glo Live Cell Substrate (Promega) diluted in Opti-MEM was added to the wells and then the plates were incubated at room temperature for 1 h in the dark, followed by measurement of cellular luciferase signal.

### ELISpot assay

ELISpot assays were performed on isolated murine splenocytes as previously described (7). Multiscreen HTS-IP filter plates (Millipore, USA) were coated with anti-mouse IFN-γ, IL-2, and IL-4 antibodies according to the manufacturer’s instructions (Mabtech, Sweden). The resuspended splenocytes were plated at a density of 5 × 10^5^ cells/well. VLPs were diluted in RPMI 1640 medium to a final concentration of 20 µg/mL and added to the plate. Negative (RPMI 1640 medium) and positive controls (Cell Stimulation Mix; Thermo Fisher Scientific) were added to each assay. Plates were incubated for 48 h at 37°C and 5% CO_2_ and then were incubated by adding biotinylated anti-mouse IFN-γ, IL-2, and IL-4 antibodies, HRP-conjugated streptavidin, and TMB substrate. Spots were counted using an Immuno-spot analyzer (Cellular Technology Ltd., USA). The results are expressed as the number of spot-forming cells in 5 × 10^5^ cells.

### Cytokine quantification using a protein array

Mouse dLNs from all groups were used for cytokine profiling, and samples from two mice in each group were mixed. The dLNs were homogenized in PBS containing protease inhibitors. Triton X-100 was then added at a final concentration of 1%. Samples were frozen at −80°C, thawed, and centrifuged at 10,000 × *g* for 5 min to remove cellular debris. The relative levels of different cytokines were assessed using the mouse cytokine array Panel A (Cat#ARY006, R&D Systems), according to the manufacturer’s instructions. Pixel density was evaluated using ImageJ Software.

### RNA-seq analysis

RNA-seq and differential expression analysis were performed at Novogene Corporation, which included read count normalization, model-dependent *P*-value estimation, and false discovery rate value estimation based on multiple hypothesis testing. This was preceded by raw read filtering, mapping clean reads to a reference genome using HISAT2, and determining the fragments per kilobase per million mapped fragments values for all samples. Differentially expressed genes were evaluated based on their log2 fold change and adjusted *P*-values. Those with |log2 (foldchange)| ≥ 1 and adjusted *P*-values ≤0.05 were considered to be differentially expressed and significant. Pathway analysis of significantly differentially expressed genes identified from the RNA-seq results was performed using reactome and KEGG in R Studio v.3.0.3. Pathways and biological processes with adjusted *P*-value ≤0.05 were considered significant.

### Splenocyte and dLN cell preparation

Mouse spleens were homogenized with a syringe plunger and passed through 40 µm cell strainers to produce single cells. Erythrocytes were lysed in lysis buffer (10 mM KHCO_3_, 150 mM NH_4_Cl, and 10 mM EDTA, pH 7.4). DLNs (popliteal, inguinal, and iliac LNs) were harvested after immunization and digested with a combination of 1 mg/mL type IV collagenase (Cat#c5138, Sigma-Alidrich) and 100 µg/mL DNAase I (Cat#D8071, Solarbio) at 37°C for 20 min, homogenized with a syringe plunger, and filtered through a 40 µm cell strainer to make a single-cell suspension. Cell viability was calculated to be 95%–100%, as determined by trypan blue exclusion.

### Flow cytometry detection

All staining steps were performed at 4°C in PBS buffer (containing 0.5% bovine serum albumin and 50 mM EDTA-2Na). Single-cell suspensions were blocked with an anti-CD16/CD32 monoclonal antibody (Thermo Fisher Scientific) and stained using the LIVE/DEAD staining kit (Invitrogen). After staining for surface markers or intracellular cytokines, all samples were fixed with 1% paraformaldehyde for 30 min, collected on a BD LSRFortessa (BD Biosciences), and analyzed using FlowJo v.10.

#### Intracellular cytokine staining

Splenocytes were plated in 96-well tissue culture plates and stimulated with GI.1 or GII.4 VLP proteins at a final concentration of 20 µg/mL for 24 h, followed by the addition of Golgi-Plugs (BD Biosciences) at 37°C with 5% CO_2_ for another 6 h. After blocking with anti-CD16/CD32 monoclonal antibody and staining with the LIVE/DEAD, cells were stained with PerCP-Cy5.5-CD3ε, APC-eFluor 780-CD4, and AF700-CD8a for 1 h and permeabilized with fixation/permeabilization solution (BD Biosciences). Samples were then intracellularly stained by incubating with APC-IFN-γ, PE-IL-2, and BV 605-IL-4 for 1 h at 4°C. The gating strategy is shown in Fig. S3a. Details of the staining panel are provided in Table S1.

#### GC B staining

After blocking with anti-CD16/CD32 monoclonal antibody and staining with LIVE/DEAD, LN cells were stained with eFlour450-IgD, FITC-GL7, PerCP-Cy5.5-CD19, PE-Cy7-Fas (CD95), and AF647 conjugated to GI.1 or GII.4 VLPs for 1 h. The gating strategy is shown in Fig. S3b. Details of the staining panel are provided in Table S2.

#### Innate immune cell staining

LN cells were divided into three panels and one panel for DCs and monocytes: BV421-CD86, BV510-CD80, BV605-PDCA-1, FITC-CD45, PerCP-Cy5.5-CD19, PE/Dazzle 594-Ly-6C, PE-Cy7-CD11b, APC-CD11c, AF700-MHCII(I-A/I-E), APC-Cy7-CD3ε; the second panel for neutrophils and macrophages: BV421-Ly6G, FITC-CD45, Percp-Cy5.5-CD19, PE-TCR-β, PE-eFluor610-CD69, PE-Cy7-CD11b, APC-Cy7-F4/80 or BV421-CD86, BV510-CD80, FITC-CD45, Percp-Cy5.5-CD19, PE-TCR-β, PE-eFluor610-CD69, PE-Cy7-CD11b, APC-Ly6G, APC-Cy7-F4/80; the third panel for lymphocytes: BV421-NK1.1, FITC-CD4, Percp-Cy5.5-CD19, PE-eFluor610- CD69, AF700-CD8a, APC-Cy7- CD3ε. After blocking with anti-CD16/CD32 monoclonal antibody and stained with the LIVE/DEAD, LN cells were stained with the above-mentioned antibodies at 4°C for 1 h. The gating strategy is presented in Fig. S3c. Details of the staining panel are provided in Tables S3 to S5.

#### BMDC staining

After blocking with anti-CD16/CD32 monoclonal antibody and staining with LIVE/DEAD, the cells were stained with BV421-CD86, BV510-CD80, FITC-CD11c, Percp-Cy5.5-MHCI (H-2Kb/H-2Db), and AF700-MHCII (I-A/I-E) for 1 h. The gating strategy is shown in Fig. S3d. Details of the staining panel are provided in Table S6.

### BMDC isolation, culture, and stimulation

BMDCs were derived from 6- to 8-week-old female BALB/c mice and cultured as described previously (66). Bone marrow cells were isolated from mouse leg bones under sterile conditions. Then, 2 × 10^6^ bone marrow cells were seeded in a 90 mm diameter culture dish in RPMI 1640 medium containing 10% fetal bovine serum, 50 µM β-mercaptoethanol, and 20 ng mL^−1^ of GM-CSF. On the 3rd day, 10 mL of medium (RPMI 1640 medium containing 10% FBS, 50 µM β-mercaptoethanol) containing GM-CSF (20 ng mL^−1^) was added. On day 6, the cells were centrifuged and resuspended in 10 mL of culture medium to continue the culture. The BMDCs were collected on day 9 for subsequent experiments.

BMDCs (1 × 10^6^ cells/well) were seeded on a 24-well plate and stimulated with 25, 50, and 100 µg/mL LNP, as well as with PMB (10 µg/mL), PMB + 100 µg/mL LNP for 24 h, and the same volume of media was added in cells as a medium control. Cells were collected for flow cytometry to detect the expression of CD80, CD86, MHC I (H-2Kb/H-2Db), and MHC II (I-A/I-E) in the CD11c^+^ population. For cytokine expression analysis, BMDCs were stimulated with 25, 50, and 100 µg/mL LNP for 8 h, and cellular RNA was extracted to measure the expression levels of cytokines using real-time PCR.

### TLR3/4/7/9 inhibition

BMDCs were seeded in a 24-well plate at a density of 1 × 10^6^ cells/well, and pretreated with TLR3 inhibitor CU CPT 4a (25 µM), TLR4 inhibitor TKA-242 (100 nM), TLR7 inhibitor (eupatorine, 10 µM), or TLR9 inhibitor ODN2088 (10 µM) for 1 h. Subsequently, the cells were stimulated with 100 µg/mL LNP for 8 h, and RNA was extracted to measure the expression levels of cytokines using real-time PCR.

### Real-time PCR

RNA was extracted from cells using an RNA isolation kit (Cat#AC0205, Sparkjade). RNA was reverse transcribed using the Hifair III 1st Strand cDNA Synthesis SuperMix (Yeasen). Real-time PCR was performed using the SYBR Premix kit (Yeasen) and analyzed using a Roche 480 Real-Time PCR System (Eppendorf, Germany). The primers used in this study are listed in Table S7.

### Statistical analysis

The Prism 9.5.1 software (GraphPad, USA) was used to perform statistical analyses. Data are shown as mean ± SEM. For multiple-group comparisons, one-way or two-way analysis of variance was used, followed by Tukey’s *post hoc* multiple comparison test. Statistical differences were considered significant when *P*-values ≤0.05.

## Data Availability

The RNA sequencing data in this study have been deposited in the Gene Expression Omnibus (GEO) database under accession number: GSE279372. All data upon which conclusions are drawn are included in the article or the supplemental material.

## SUPPLEMENTAL MATERIAL

Supplemental material jvi.01699-24-s0001.pdf Figures S1 to S4; Tables S1 to S7.

ASM does not own the copyrights to Supplemental Material that may be linked to, or accessed through, an article. The authors have granted ASM a non-exclusive, world-wide license to publish the Supplemental Material files. Please contact the corresponding author directly for reuse.
