# Supplementary material for: Lipid nanoparticles as adjuvant of norovirus VLP vaccine augment cellular and humoral immune responses in a TLR9- and type I IFN-dependent pathway
Source: J Virol. 2024 Nov 4;98(12):e01699-24. doi: 10.1128/jvi.01699-24 (PMC11650981; doi:10.1128/jvi.01699-24)
Supplement: Supplemental material — Figures S1 to S4; Tables S1 to S7. [file jvi.01699-24-s0001.pdf]

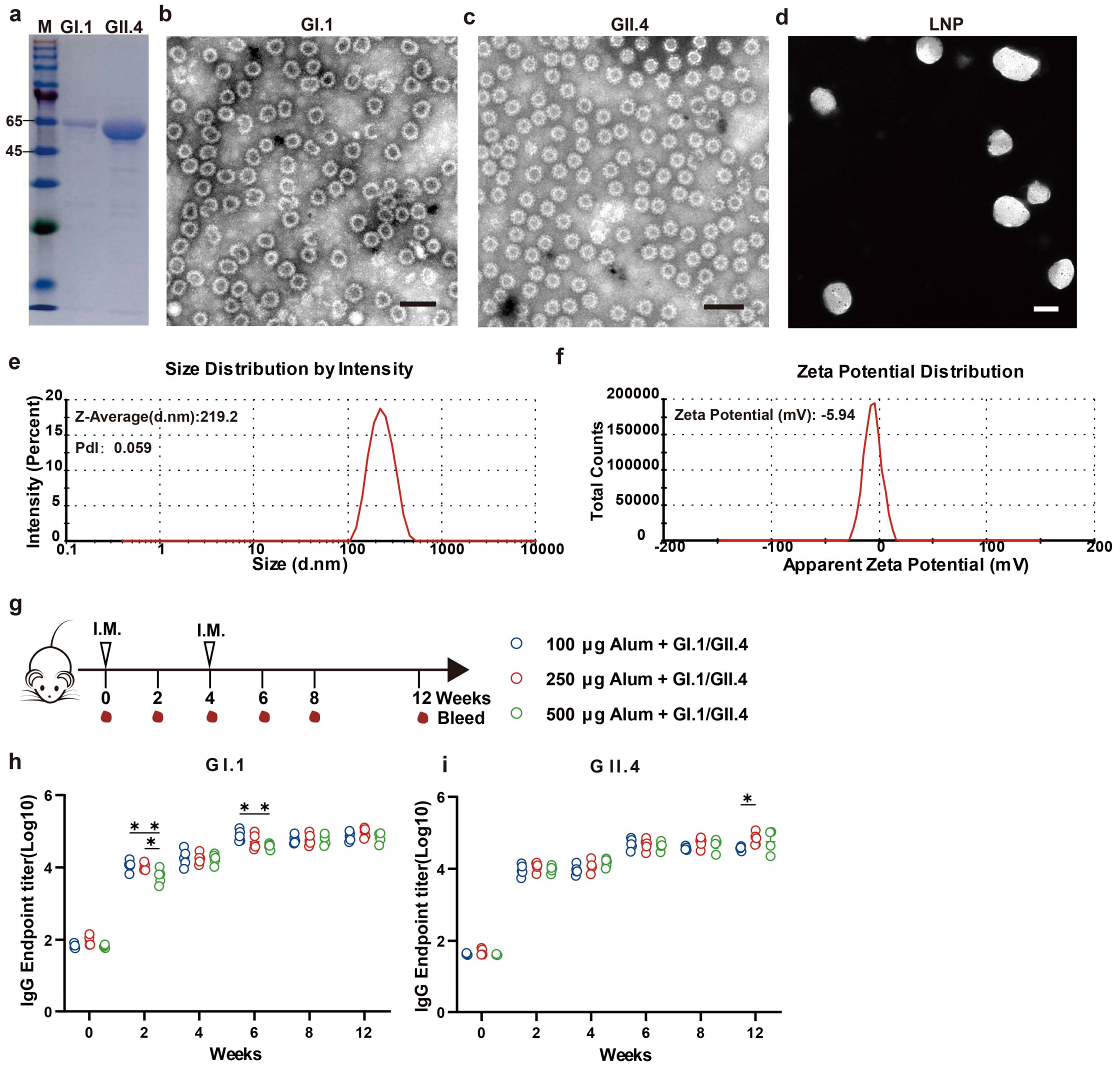

FIG S1 Characterization of GI.1 and GII.4 VLPs and LNPs and Alum adjuvant dose optimization experiments. (**a**) SDS-PAGE of GI.1 and GII.4 VLPs. TEM images of GI.1 (**b**) and GII.4 VLPs (**c**). Scale bars represent 100 nm. (**d**) The TEM image of LNPs. The scale bar represents 200 nm. The size (**e**) and Zeta potential (**f**) of LNPs. (**g**) BALB/c mice (*n* = 5/group) received two i.m. immunizations at weeks 0 and 4. Then, 5 μg of each GI.1 and GII.4 VLPs were mixed with 100 μg, 250 μg or 500 μg Alum separately. Sera were collected at weeks 0, 2, 4, 6, 8 and 12 post-priming. Binding IgG against (**h**) GI.1 and (**I**) GII.4 VLPs were determined by ELISA. Data are shown as mean ± SEM. (Two-way ANOVA with Tukey's multiple comparisons. $^{*}P < 0.05$, $^{**}P < 0.01$).

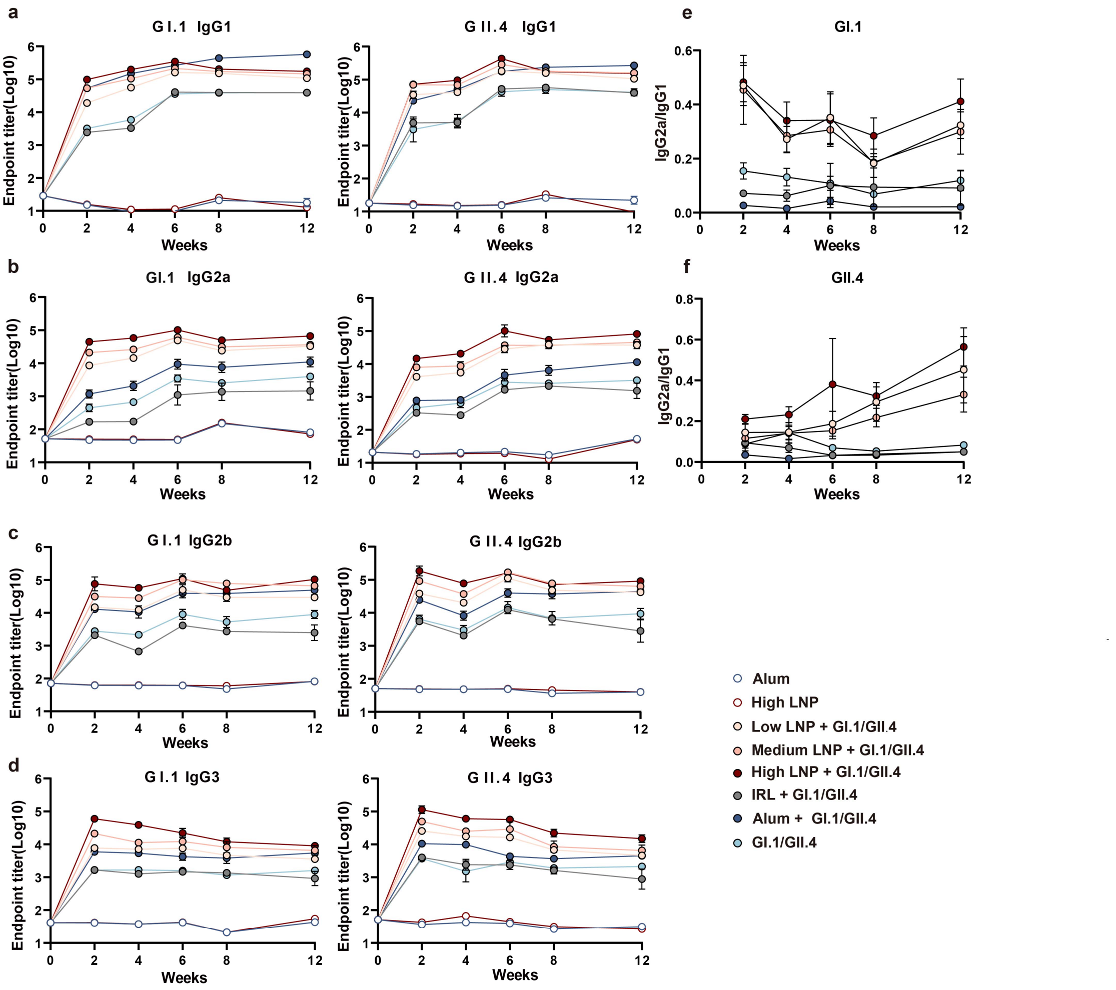

FIG S2 Detection of IgG subclasses in mice with different immunization groups. VLP-specific IgG1 (**a**), IgG2a (**b**), IgG2b (**c**), and IgG3 (**d**) for 0–12 weeks were detected by ELISA. GI.1 VLP-specific antibodies were displayed on the left and GII.4 VLP-specific antibodies were displayed on the right. (**e**) GI.1 VLP IgG2a/IgG1 ratios. (**f**) GII.4 VLP IgG2a/IgG1 ratios. Data are shown as the mean ± SEM.

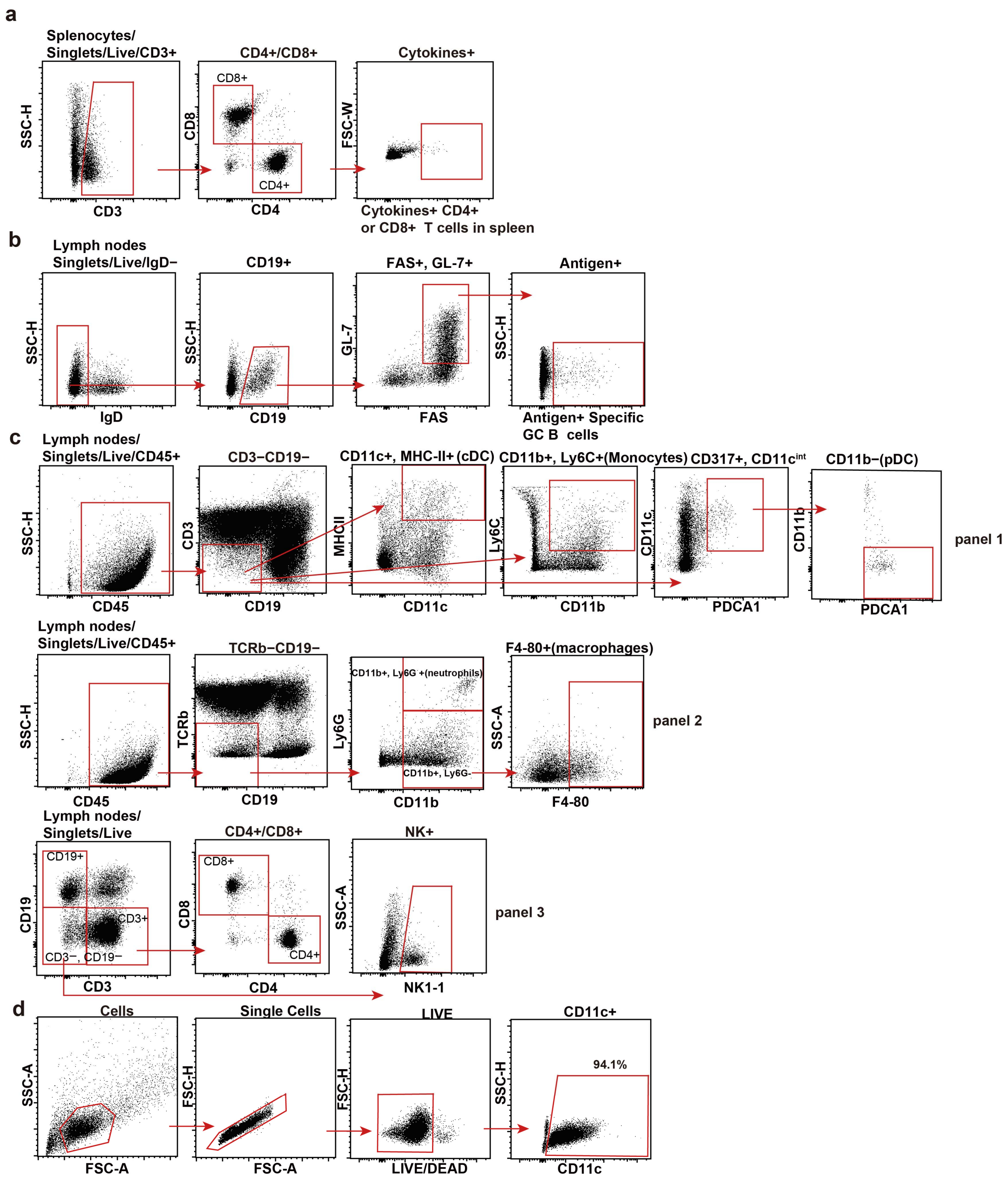

FIG S3 Gating strategy for measuring different cell types. (**a**) Gating strategy of splenic CD8$^+$ T cells and CD4$^+$ T cells, which were pre-gated on singlet live cells. Cytokine-secreted T cell populations were gated downstream, respectively. (**b**) Gating strategy for measuring total GC B (IgD$^-$ CD19$^+$ Fas$^+$ GL7$^+$) and antigen$^+$ GC B (IgD$^-$ CD19$^+$ Fas$^+$ GL7$^+$ antigen$^+$) populations in dLNs, which were pre-gated on singlet live cells. (**c**) Gating strategy for measuring cDC (CD45$^+$ CD3$^-$ CD19$^-$ CD11c$^+$ MHCII$^+$), monocytes (CD45$^+$ CD3$^-$ CD19$^-$ CD11b$^+$ Ly6C$^+$), pDC (CD45$^+$ CD3$^-$ CD19$^-$ CD317$^+$ CD11C^{int} CD11b$^-$), macrophages (CD45$^+$ TCRβ$^-$ CD19$^-$ CD11b$^+$ Ly6G$^-$ F4/80$^+$), neutrophils (CD45$^+$ TCRβ$^-$ CD19$^-$ CD11b$^+$ Ly6G$^+$), CD4$^+$, CD8$^+$, and NK cells (CD3$^-$ CD19$^-$ NK1.1$^+$) in dLNs, which were pre-gated on singlet live cells. (**d**) Representative dot plots of BMDC(FSC/SSC/Single/Live/CD11c$^+$).

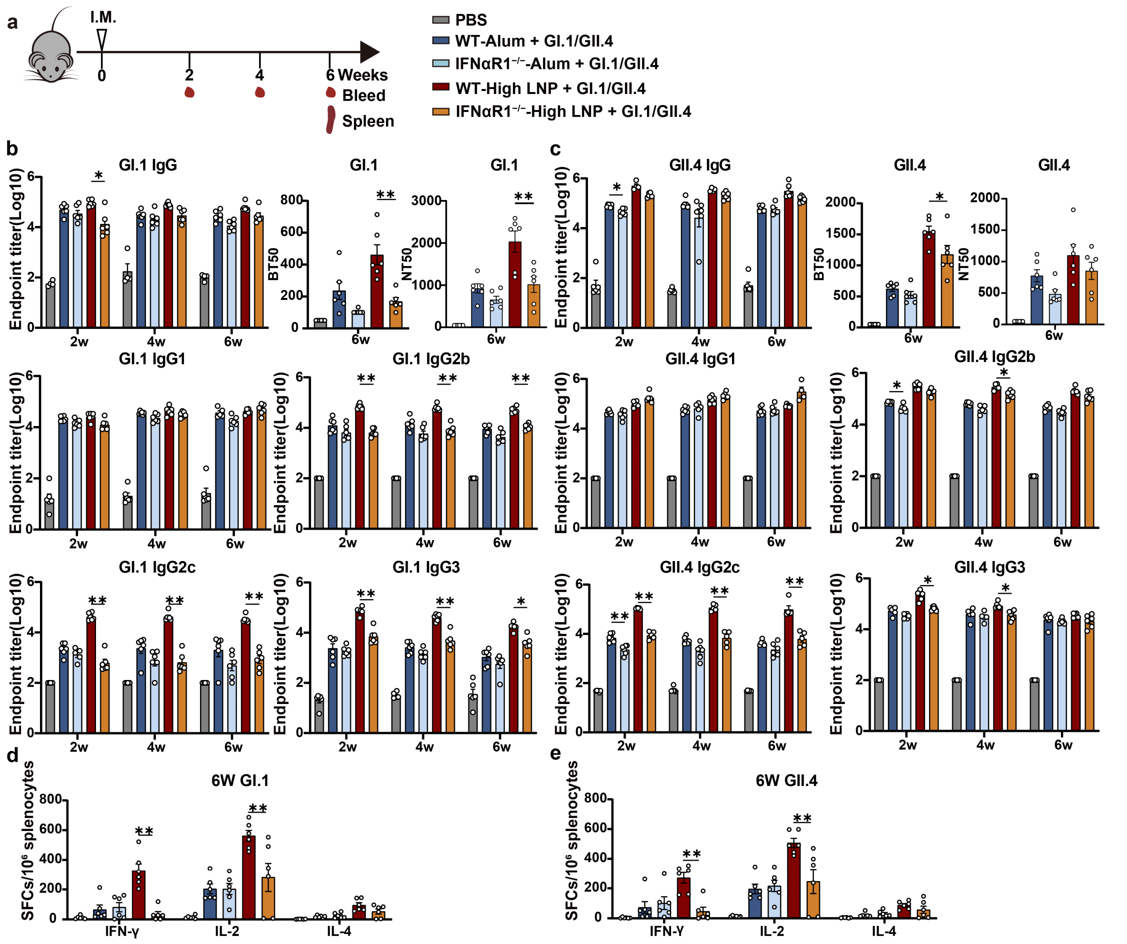

FIG S4. IFNαR1 knockout impacts cellular and humoral immune responses in mice. (**a**) Immunization schedule. C57BL/6J WT and IFNαR1$^{-/-}$ mice were injected i.m. with High LNP + GI.1/GII.4 VLP or Alum + GI.1/GII.4 VLP, and WT mice were inoculated with PBS as a control group (n = 6/group). GI.1 VLP (**b**) and GII.4 VLP (**c**) specific-binding antibodies, blocking antibodies, neutralizing antibodies, and IgG subclasses were detected. ELISpot was used to detect the ability of splenocytes to release IFN-γ, IL-2, and IL-4 after restimulation with GI.1 (**d**) or GII.4 VLP (**e**) VLP at week 6. Data are shown as mean ± SEM. All data show statistical analysis between C57BL/6J WT and IFNaR1$^{-/-}$mice for each vaccinated group. (One-way ANOVA test with Tukey's multiple comparisons test for NT50 and BT50 in **b-c**. Two-way ANOVA and Tukey's multiple comparisons test for the other data in **b-e**. $^{*}P < 0.05$; $^{**}P < 0.01$).

Table S1 Intracellular Cytokine Staining panel

Stain	Fluorochrome	Clone	Vendor	Catalogue #
CD3ε	PerCP-Cy5.5	145-2C11	BioLegend	100328
CD4	APC-eFluor™ 780	GK1.5	eBioscience	47-0041-82
CD8a	AF 700	53-6.7	eBioscience	56-0081-82
IFN-γ	APC	XMG1.2	BioLegend	505810
IL-2	PE	JES6-5H4	eBioscience	12-7021-82
IL-4	BV 605	11B11	BioLegend	504125

Table S2 GC B staining cell panel

Stain	Fluorochrome	Clone	Vendor	Catalogue #
IgD	eFlour450	11-26c	Invitrogen	48-5993-82
GL7	FITC	GL7	BioLegend	144604
CD19	PerCP-Cy5.5	6D5	BioLegend	115534
Fas (CD95)	PE-Cy7	Jo2	BD Biosciences	557653
GI.1	AF647	-	Labeled in-house	ab269823
GII.4	AF647	-	Labeled in-house	ab269823

Table S3 Innate immune cell staining panel 1 (DCs and monocytes)

Stain	Fluorochrome	Clone	Vendor	Catalogue #
CD86	BV421	GL-1	BioLegend	105031
CD80	BV510	16-10A1	BioLegend	104741
PDCA-1	BV605	927	BioLegend	127025
CD45	FITC	S18009F	BioLegend	157214
CD19	PerCP-Cy5.5	6D5	Biolegend	115534
Ly-6C	PE/Dazzle™ 594	HK1.4	BioLegend	128043
CD11b	PE-Cy7	M1/70	BioLegend	101215
CD11c	APC	N418	BioLegend	117310
MHCII(I-A/I-E)	AF700	M5/114.15.2	eBioscience	56-5321-82
CD3ε	APC-Cy7	500A2	Biolegend	152324

Table S4 Innate immune cell staining panel 2 (Neutrophils and macrophages)

Stain	Fluorochrome	Clone	Vendor	Catalogue #
Ly6G	BV421	1A8	BD Biosciences	562737
CD45	FITC	S18009F	BioLegend	157214
CD19	Percp-Cy5.5	6D5	Biolegend	115534
TCR-β	PE	H57-597	BioLegend	109207
CD69	PE-eFluor610	H1.2F3	Invitrogen	61-0691-80
CD11b	PE-Cy7	M1/70	BioLegend	101215
F4/80	APC-Cy7	BM8	BioLegend	123117
CD86	BV421	GL-1	BioLegend	105031
CD80	BV510	16-10A1	BioLegend	104741
Ly6G	APC	1A8	Biolegend	127613

Table S5 Innate immune cell staining panel 3 (Lymphocytes)

Stain	Fluorochrome	Clone	Vendor	Catalogue #
NK1.1	BV421	PK136	BioLegend	108741
CD4	FITC	RM4-5	Biolegend	100510
CD19	Percp-Cy5.5	6D5	Biolegend	115534
CD69	PE-eFluor610	H1.2F3	Invitrogen	61-0691-80
CD8a	AF 700	53-6.7	eBioscience	56-0081-82
CD3ε	APC-Cy7	500A2	Biolegend	152324

Table S6 BMDC staining

Stain	Fluorochrome	Clone	Vendor	Catalogue #
CD86	BV421	GL-1	BioLegend	105031
CD80	BV510	16-10A1	BioLegend	104741
CD11c	FITC	N418	BioLegend	117305
MHCI(H-2Kb/H-2Db)	Percp-Cy5.5	28-8-6	BioLegend	114619
MHCII(I-A/I-E)	AF700	M5/114.15.2	eBioscience	56-5321-82

Table S7 Primer sequences

Gene	Forward sequence (5'-3')	Reverse sequence (5'-3')
Actin	AGTGTGACGTTGACATCCGT	GCAGCTCAGTAACAGTCCGC
CXCL10	GCCGTCATTTTCTGCCTCA	CGTCCTTGCGAGAGGGATC
IFN-α1	GCCTTGACACTCCTGGTACAAATGA	CAGCACATTGGCAGAGGAAGACAG
IFN-α4	TGATGAGCTACTACTGGTCAGC	GATCTCTTAGCACAAGGATGGC
IFN-β1	CAGCTCCAAGAAAGGACGAAC	GGCAGTGTAACTCTTCTGCAT
IRF-7	GGGAGGCCCAAGGAGAAG	CATACCCATGGCTCCAGCTT
IFIT3	GCTCAGGCTTACGTTGACAAGG	CTTTAGCGTGTCCATCCTTCC
IFN-γ	CAGCAACAGCAAGGCGAAAAAGG	TTTCCGCTTCCTGAGGCTGGAT
